# Questions and controversies in the study of time-varying functional connectivity in resting fMRI

**DOI:** 10.1162/netn_a_00116

**Published:** 2020-02-01

**Authors:** Daniel J. Lurie, Daniel Kessler, Danielle S. Bassett, Richard F. Betzel, Michael Breakspear, Shella Kheilholz, Aaron Kucyi, Raphaël Liégeois, Martin A. Lindquist, Anthony Randal McIntosh, Russell A. Poldrack, James M. Shine, William Hedley Thompson, Natalia Z. Bielczyk, Linda Douw, Dominik Kraft, Robyn L. Miller, Muthuraman Muthuraman, Lorenzo Pasquini, Adeel Razi, Diego Vidaurre, Hua Xie, Vince D. Calhoun

**Affiliations:** Department of Psychology, University of California, Berkeley, Berkeley, CA, USA; Departments of Statistics and Psychiatry, University of Michigan, Ann Arbor, MI, USA; Department of Bioengineering, School of Engineering and Applied Sciences, University of Pennsylvania, Philadelphia, PA, USA; Department of Physics & Astronomy, College of Arts & Sciences, University of Pennsylvania, Philadelphia, PA, USA; Department of Neurology, Perelman School of Medicine, University of Pennsylvania, Philadelphia, PA, USA; Department of Electrical & Systems Engineering, School of Engineering and Applied Sciences, University of Pennsylvania, Philadelphia, PA, USA; Department of Bioengineering, School of Engineering and Applied Sciences, University of Pennsylvania, Philadelphia, PA, USA; University of Newcastle, Callaghan, NSW, 2308, Australia; QIMR Berghofer, Brisbane, Australia; Department of Biomedical Engineering, Emory University and Georgia Institute of Technology, Atlanta, GA, USA; Department of Neurology and Neurological Sciences, Stanford University, Stanford CA, USA; Institute of Bioengineering, Center for Neuroprosthetics, Ecole Polytechnique Fédérale de Lausanne, Switzerland; Department of Radiology and Medical Informatics, University of Geneva, Switzerland; Department of Biostatistics, Johns Hopkins University, Baltimore, MD, USA; Rotman Research Institute - Baycrest Centre, Toronto, Canada; Department of Psychology, University of Toronto, Toronto, Canada; Department of Psychology, Stanford University, Stanford, CA, USA; Brain and Mind Centre, University of Sydney, NSW, Australia; Department of Psychology, Stanford University, Stanford, CA, USA; Department of Clinical Neuroscience, Karolinska Institutet, Stockholm, Sweden; Stichting Solaris Onderzoek en Ontwikkeling, Nijmegen, The Netherlands; Department of Anatomy and Neurosciences, VU University Medical Center, Amsterdam, The Netherlands; Department of Psychology, Goethe University Frankfurt, Frankfurt am Main, Germany; The Mind Research Network, Albuquerque, NM, USA; Biomedical Statistics and Multimodal Signal Processing Unit, Movement Disorders and Neurostimulation, Department of Neurology, Focus Program Translational Neuroscience, Johannes-Gutenberg-University Hospital, Mainz, Germany; Memory and Aging Center, Department of Neurology, University of California, San Francisco, San Francisco, CA, USA; Monash Institute of Cognitive and Clinical Neurosciences and Monash Biomedical Imaging, Monash University, Clayton, Australia; Wellcome Centre for Human Neuroimaging, Institute of Neurology, University College London, London, United Kingdom; Department of Electronic Engineering, NED University of Engineering and Technology, Karachi, Pakistan; Wellcome Trust Centre for Integrative Neuroimaging, Oxford Centre for Human Brain Activity, University of Oxford, United Kingdom; Department of Psychiatry and Behavioral Sciences, Stanford University, Stanford, CA, USA; The Mind Research Network, Albuquerque, NM, USA; Department of Electrical and Computer Engineering, University of New Mexico, Albuquerque, NM, USA; Tri-institutional Center for Translational Research in Neuroimaging and Data Science (TReNDS), Georgia State, Georgia Tech, Emory, Atlanta, Georgia, USA

**Keywords:** Functional connectivity, Brain networks, Brain dynamics, fMRI, Rest, Review

## Abstract

The brain is a complex, multiscale dynamical system composed of many interacting regions. Knowledge of the spatiotemporal organization of these interactions is critical for establishing a solid understanding of the brain’s functional architecture and the relationship between neural dynamics and cognition in health and disease. The possibility of studying these dynamics through careful analysis of neuroimaging data has catalyzed substantial interest in methods that estimate time-resolved fluctuations in functional connectivity (often referred to as “dynamic” or time-varying functional connectivity; TVFC). At the same time, debates have emerged regarding the application of TVFC analyses to resting fMRI data, and about the statistical validity, physiological origins, and cognitive and behavioral relevance of resting TVFC. These and other unresolved issues complicate interpretation of resting TVFC findings and limit the insights that can be gained from this promising new research area. This article brings together scientists with a variety of perspectives on resting TVFC to review the current literature in light of these issues. We introduce core concepts, define key terms, summarize controversies and open questions, and present a forward-looking perspective on how resting TVFC analyses can be rigorously and productively applied to investigate a wide range of questions in cognitive and systems neuroscience.

## TIME-VARYING FUNCTIONAL CONNECTIVITY: AN INTRODUCTION

Even when sitting quietly in a dark room, the brain is active, yielding a constant stream of thoughts and ideas, along with changes in awareness, arousal, and vigilance. The brain constantly constructs and updates internal models of the world to anticipate and plan future adaptive behaviors (Parr, Rees, & Friston, 2018), and wakeful rest is no less cognitively rich and complex than task engagement. The notion that patterns of neuronal activity and interregional coupling may exhibit the statistical and dynamical fingerprints of these mental wanderings—even in the absence of an explicit task—accords with the most fundamental observations of our “stream of consciousness.” While it is relatively straightforward to quantify changes in brain activity and functional connectivity that are time-locked to perceptual stimuli and externally cued tasks (Cohen, 2018; Gonzalez-Castillo & Bandettini, 2018), detecting and characterizing changes that arise “spontaneously”—from endogenous and unknown causes and at seemingly random times—is substantially more difficult. Despite these challenges, studies of intrinsic brain dynamics and self-directed “resting”cognition provide an important, ecologically valid perspective on brain function and mental life. A large proportion of our time (up to 50%) is spent engaging in cognition and behavior unrelated to the task at hand (Killingsworth & Gilbert, 2010), and emerging evidence suggests that these task-unrelated thoughts and actions may explain up to twice the variance in neural activity than task-related variables (Musall, Kaufman, Juavinett, Gluf, & Churchland, 2019).

**Figure 1.**
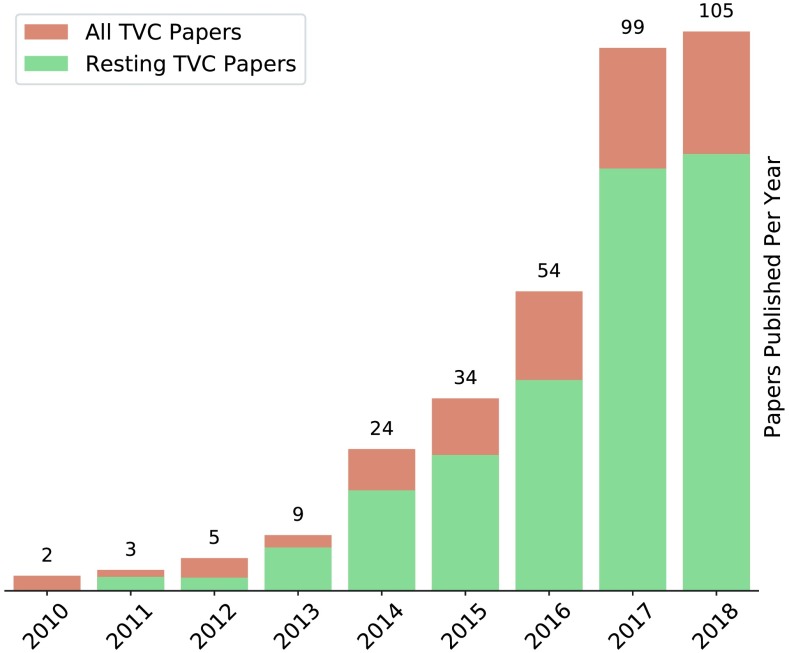
Growth of the fMRI TVFC literature. The field of TVFC research has grown rapidly, as demonstrated by the increasing number of fMRI TVFC papers published each year (as indexed by PubMed). To account for overall growth in the rate of scientific publishing, the height of the bars has been normalized by the total number of all papers published in each year. Because of inconsistencies in the way TVFC analyses are described, these figures likely represent a conservative estimate of the size of the fMRI TVFC literature, particularly for earlier years. For details on the search terms used to identify TVFC papers, please see the Supporting Information.

Functional connectivity (FC) analyses of resting fMRI (rfMRI) data allow researchers to noninvasively estimate patterns of interregional neural interactions. An integral component of modern neuroimaging research, FC is traditionally calculated over an entire scan or experimental condition (“static” functional connectivity), but recent years have seen rapidly growing interest in studying time-resolved fluctuations in FC (often referred to as “dynamic” or time-varying functional connectivity; TVFC; Calhoun, Miller, Pearlson, & Adali, 2014; Hutchison, Womelsdorf, Allen, et al., 2013; see Figure 1). A burgeoning literature now spans studies using varied imaging modalities (e.g., fMRI, Sakoglu et al., 2010; EEG, Tagliazucchi, von Wegner, Morzelewski, Brodbeck, & Laufs, 2012; and MEG, Baker et al., 2014) to investigate fluctuations in FC during a wide range of cognitive and behavioral states ranging from explicitly cued task execution (e.g., Gonzalez-Castillo & Bandettini, 2018) to wakeful rest (e.g., Allen et al., 2014), sleep (e.g., Tagliazucchi & Laufs, 2014), and anesthesia (e.g., Hutchison, Womelsdorf, Gati, Everling, & Menon, 2013). Interindividual differences in resting TVFC have been associated with a wide range of cognitive and behavioral traits (Liegeois et al., 2019; Vidaurre, Smith, & Woolrich, 2017), and emerging evidence suggests that in some cases TVFC may be a more sensitive marker of these differences than static FC (Jin et al., 2017; Liegeois et al., 2019; Rashid et al., 2016; Vidaurre, Llera, Smith, & Woolrich, 2019). Alterations in TVFC have also been observed in a growing number of psychiatric and neurological conditions including autism (de Lacy, Doherty, King, Rachakonda, & Calhoun, 2017), ADHD (de Lacy & Calhoun, in press), depression (Kaiser et al., 2016), PTSD (Jin et al., 2017), schizophrenia (Sakoglu et al., 2010), Parkinson’s (Diez-Cirarda et al., 2018), and Alzheimer’s disease (Jones et al., 2012).

Like any emerging research program, resting TVFC research has encountered its share of growing pains and challenges. Studying the brain at rest has a number of advantages—minimal demands on study participants, analytic flexibility afforded by the lack of an externally imposed task, the absence of potential performance confounds—and may potentially provide a richer characterization of brain activity than task studies (Ponce-Alvarez, He, Hagmann, & Deco, 2015). However, while resting TVFC research benefits from the advantages of rfMRI, it also suffers from its pitfalls: the lack of clear benchmarks, the absence of experimental control of behavioral or cognitive state, and the inability to objectively monitor behavioral task performance. Paralleling similar debates from the early days of rfMRI (see Box 1), there is active debate about the extent to which BOLD TVFC is able to detect transient changes in neural signaling or cognitive state during rest. A number of important open questions contribute to this lack of consensus: To what extent are estimates of resting BOLD TVFC driven by fluctuations in arousal and cognitive state versus nonneural physiological factors (e.g., head motion, cardiovascular and respiratory effects)? What are the most appropriate ways to test observed estimates of TVFC against “static” null hypotheses? Whereas detecting change-points or fluctuating dependence structure in neuroimaging data is in principle an achievable outcome of signal analysis—and indeed these are the goals of many TVFC analysis methods—understanding the putative causes of these changes requires other techniques: online measures of cognitive and bodily states, insights from pathological conditions, the inversion of generative models, and causal manipulations such as brain stimulation and administration of pharmacological agents. It is our goal to summarize the current literature surrounding these and related issues, and to provide suggestions for future work that may help adjudicate these debates.

Box 1. A brief history of studying the brain at rest Studying the brain at rest is not a new idea. Scientists have been interested in the dynamics of resting cognition at least since the writings of William James in the late 1800s (James, 1890), and much of Hans Berger’s pioneering EEG research in the 1920s was focused on the properties of intrinsic brain activity (Karbowski, 1990). Following the development of PET and BOLD fMRI in the 1980s and 1990s, human functional neuroimaging was initially dominated by task activation paradigms. However, researchers quickly began to notice a set of regions that consistently deactivated in response to external task demands, and that exhibited high metabolic activity during rest. This set of regions was named the default mode network (DMN) in a seminal 2001 paper by Raichle et al. (2001). In a complementary line of work, Biswal et al. estimated BOLD fMRI functional connectivity between primary motor cortex and other brain areas, independent of any overt task (Biswal, Yetkin, Haughton, & Hyde, 1995). The resulting spatial patterns of FC mirrored patterns of activation seen when subjects executed a motor response. These and other findings led to renewed interest in the study of the brain at rest, with the hope that better characterizing “resting state” FC networks would reveal core features of the brain’s functional organization.

Neuroimaging studies of the brain at rest quickly converged on a set of canonical FC networks that are consistently observed at rest and correspond with patterns of task-evoked activation and functional connectivity (Calhoun, Kiehl, & Pearlson, 2008; Damoiseaux et al., 2006; Smith et al., 2009). While early studies focused on investigating the FC of individual networks (e.g., DMN; M. D. Greicius, Krasnow, Reiss, & Menon, 2003), this work eventually expanded into efforts to investigate global functional organization by mapping FC across the whole brain (e.g., Yeo et al., 2011). These initial observations have been widely replicated across hundreds of studies using a variety of analytic methods (e.g., seed-based functional connectivity, ICA, community detection).

Interindividual differences in resting FC patterns have been associated with a wide range of phenotypic traits (e.g., working memory and executive control; Cole, Yarkoni, Repovs, Anticevic, & Braver, 2012; Hampson, Driesen, Skudlarski, Gore, & Constable, 2006) and clinical conditions (e.g., psychiatric and neurological disorders; Fox & Greicius, 2010; M. Greicius, 2008), and can be used to predict behavioral performance (e.g., M. D. Rosenberg et al., 2016) and individual identity (e.g., Finn et al., 2015).

Despite the success of the resting FC research program in expanding our understanding of human brain function, it has historically been limited by the use of methods that are unable to address fundamental motivating questions about inherently dynamic cognitive and neural processes. In response to this limitation, the past decade has seen the emergence of new tools for studying the time-varying properties of the brain at rest.

While there are indeed real points of fundamental disagreement among researchers about various aspects of BOLD TVFC, debates in the literature have at times been needlessly muddied by inconsistent or imprecise definitions and operationalizations. For example, the term “metastate” has been variously used to describe (a) a small number of replicable patterns of connectivity that recur across or within individuals (i.e., functional connectivity states; Shine, Koyejo, & Poldrack, 2016), (b) subsets of functional connectivity and activity states that share certain temporal characteristics (Vidaurre et al., 2017), or (c) a specific location in a second-order state-space (R. L. Miller et al., 2016). As has been previously suggested (Liegeois, Laumann, Snyder, Zhou, & Yeo, 2017; R. L. Miller, Abrol, Adali, Levin-Schwarz, & Calhoun, 2018), we believe that progress on resolving these debates requires standardizing our terminology and identifying common frameworks. While intuitive notions of brain dynamics may seem straightforward, there is currently no consensus about operational definitions for many key concepts related to TVFC. Establishing appropriate terminology for the phenomenon under study is particularly important. Although “dynamic functional connectivity” is frequently used in the literature, different uses and definitions of the term “dynamic” across disciplines can lead to troublesome ambiguity. As such, we have opted here to use the more broadly applicable phrase “time-varying functional connectivity,” where functional connectivity refers to any of various notions of statistical dependence, most commonly (but not exclusively) correlation between time series. We define this and other key terms in the glossary presented in Table 1, while Box 2 provides a brief discussion of the nuances involved in relating TVFC estimates to the underlying neural phenomena we seek to study and understand.

**Table 1.** Glossary of key terms

Term	Definition
**Functional connectivity (FC)**	Statistical dependencies among neurophysiological time series derived from regions or networks. Most often estimated as a correlation coefficient.

**Static functional connectivity**	An estimate of statistical dependence made under the assumption that the dependence structure does not vary as a function of time.

**Statistical stationarity**	A formal definition of certain statistical properties being invariant to a shift in time. In practice, stationarity can only be assessed given multiple realizations of a time series (rather than for a single dataset). – Strong stationarity: The probability distribution of the time series is invariant under a shift in time. – Weak stationarity (or second-order stationarity): The mean and covariance of the time series are finite and invariant under a shift in time. This is the definition most time series models use in practice.

**Time-varying functional connectivity (TVFC)**	Functional connectivity that varies as a function of time. Also referred to as “dynamic functional connectivity.”

**Functional connectivity state**	A transient pattern of whole-brain functional connectivity. Usually identified by analytic techniques that attempt to model the full repertoire of functional connectivity patterns as being made up of a relatively small number of FC states (often referred to in shorthand simply as “states”). Some of these low-dimensional models constrain the brain to be in a single state at a time, whereas others permit each time point to be a mixture of states.

**Activity state**	A transient pattern of whole-brain activation, analogous to a functional connectivity state.

**Windowed functional connectivity**	Functional connectivity estimated over a defined time window that is shorter than the full time series. Windowing can involve weighting or tapering. “Sliding window” methods can be used to produce time-resolved estimates of functional connectivity (one for each window).

**Dynamical system**	A system composed of interacting components (neurons, brain regions, etc.) whose state evolves forward in time according to a particular rule (such as a difference or differential equation). Such systems yield complex behaviors that can be observed via an (often indirect) measurement process.

**Hidden Markov model (HMM)**	A statistical model wherein observed data are assumed to be generated from a process that moves among unobserved states. Fitting an HMM involves estimating (1) the properties of each state, (2) transition probabilities between the states, and (3) which state is active at each time point. For TVFC applications, each state might correspond to a distinct pattern of brain activity and functional connectivity, the transition probabilities would explain how the brain moves from one state to another, and the estimates of active states would give time-resolved estimates of which state was active at each time point.

Box 2. Distinguishing the map from the territory in TVFC research When studying TVFC (and FC in general), it is critically important to ensure that one distinguishes between the method (e.g., functional connectivity operationalized as statistical dependence between time series) and the target theoretical properties we wish to infer (e.g., interregional neural interactions). Failure to do so commits the logical fallacy of confusing the map for the territory (Korzybski, 1933), and (to use a recent example from Reid et al., 2019) “is akin to defining the moon as the photons that hit one’s retina when looking at a particular location in the sky (a common method for detecting the moon), rather than as a physical object with a variety of properties consistent with the laws of physics (theoretical properties of interest)” (p. 1751).

In the context of FC, it is relatively straightforward to define the map as estimates of statistical dependence between neurophysiological time series. This definition can be extended to the case of TVFC by allowing these estimates to vary over time. Following Reid and colleagues, we define the territory as time-varying patterns of causal interaction between neural entities (e.g., neurons, populations, networks). These interactions have many different properties we may be interested in, such as their direction (e.g., A→B, B→A, etc.), directness (i.e., mono- or multisynaptic), and timing (i.e., when an interaction between two entities takes place). While most FC methods applied to BOLD fMRI data are limited in the extent to which they can provide information about the exact structure of the underlying causal graph, they nonetheless constrain the space of possible network configurations (Reid et al., 2019). Studies of TVFC extend the FC paradigm by allowing researchers to make inferences about how this space of possible network configurations changes over time, and how these changes relate to cognition and behavior.

Given the somewhat controversial nature of FC research (Mehler & Kording, 2018), it is also worth emphasizing that FC estimates (both static and time-varying) can be useful above and beyond any mechanistic information they may (or may not) provide about interregional neural interactions and their relationship to cognition. As mentioned above, patterns of FC and TVFC are sensitive to individual differences in health and disease, and emerging work suggests they may have powerful utility as clinical biomarkers (e.g., as predictors of treatment response; Drysdale et al., 2017; Etkin et al., 2019; Reggente et al., 2018).

This paper is the result of a collaborative, open-invitation community effort to review the current resting TVFC literature and to discuss key open questions and outstanding controversies regarding this exciting new domain of research. As a group of scientists with diverse perspectives on TVFC, we have attempted to reconcile and synthesize our views on controversial issues, and to contextualize them in light of alternative opinions held by others in the community. While we offer some general suggestions for how researchers might best take advantage of the TVFC research program, we avoid making specific technical or methodological recommendations except in cases where they are supported by the empirical literature.

We frame our discussion in terms of three broad questions: (1) Are rfMRI time series statistically consistent with functional connectivity that truly varies in time? (2) What is the biological basis of BOLD TVFC (neural or otherwise)? (3) What (if any) is the cognitive and behavioral relevance of resting BOLD TVFC? We begin with a survey of the current landscape of analytic and modeling approaches for studying BOLD TVFC, and then proceed to address each of the three questions outlined above. First, we review methodological considerations and statistical challenges for studying TVFC in fMRI. Second, we review the literature on the physiological basis of BOLD TVFC. Third, we provide an in-depth discussion of the cognitive and behavioral relevance of BOLD TVFC, including evidence both for and against this proposition. Subsequent sections highlight experimental approaches that may help adjudicate questions about the cognitive relevance of TVFC, and briefly review strategies for cleaning rfMRI data to mitigate the impact of potential confounds on TVFC analyses. We conclude by suggesting ways that the TVFC research community can continue to advance this exciting field and help facilitate consensus on controversial issues.

## ANALYTIC APPROACHES

Approaches to studying functional connectivity in fMRI data can be considered along a spectrum of temporal resolution. On one end, some methods assume that the dependence structure (functional connectivity) between regions is constant over an arbitrarily long time window (i.e., “static” FC); on the other end are methods that can estimate time-resolved FC at each individual time point (e.g., instantaneous and sliding-window approaches). In between are methods that aim to discover discrete, temporally contiguous functional connectivity states characterized by their interregional dependence structure (e.g., sliding windows + clustering). In these state-based models, the dependence structure changes only when moving *between* states.

Another important property of methods used to study TVFC is the extent to which they consider the temporal ordering of the observed data points. Some approaches directly leverage the information in this ordering (e.g., time-frequency approaches; Chang & Glover, 2010; Yaesoubi, Allen, Miller, & Calhoun, 2015), while others ignore ordering completely and treat data points as exchangeable samples (Liu, Zhang, Chang, & Duyn, 2018; Yaesoubi, Adali, & Calhoun, 2018). Many common TVFC analysis pipelines have stages that alternately leverage and neglect temporal ordering. For example, one might begin by estimating sliding-window correlations (calculated using time series with time points ordered as observed), apply k-means clustering to the resulting time-resolved FC matrices (k-means ignores the temporal ordering of the windows), and then evaluate state properties such as dwell times and transition probabilities (which again considers the temporal order of time points; Allen et al., 2014).

**Figure 2.**
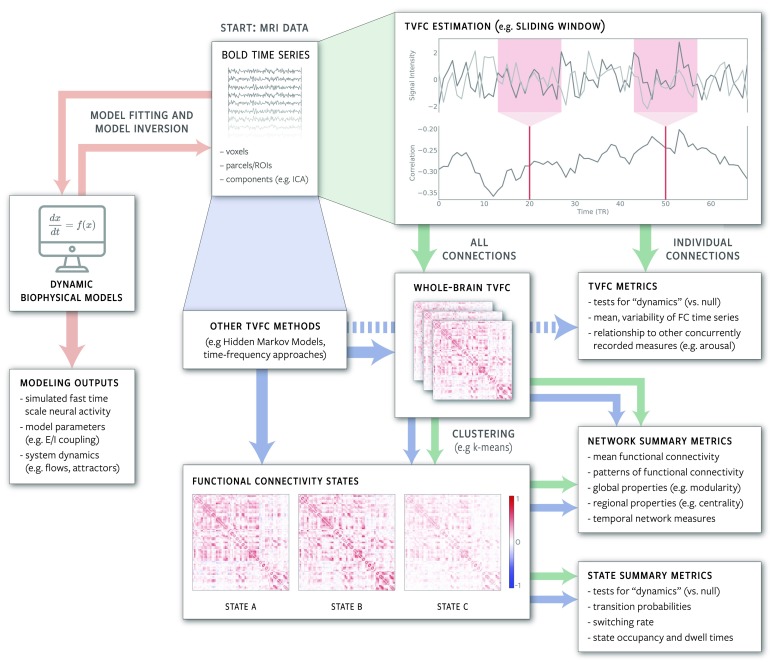
Schematic illustration of common analysis and modeling approaches for studying TVFC in fMRI data. Green arrows indicate a typical workflow based on sliding-window correlation, which is currently the most common data-driven approach for estimating TVFC. Blue arrows represent the diversity of alternative data-driven approaches. Some alternative approaches (e.g., HMMs) estimate functional connectivity states directly from BOLD time series, while others (e.g., phase synchrony, a time-frequency method) are more similar to the sliding-window approach. Regardless of how FC time series or functional connectivity states are estimated, it is possible to calculate a wide range of measures describing their properties. For example, fluctuations in the strength of FC between two areas can be tested for associations with concurrently measured behavioral variables, while network measures can be used to describe the properties of whole-brain FC patterns and how they change over time. Whether TVFC estimates are considered to constitute bona fide “dynamics” depends on the specific feature of interest and null model against which they are tested. Orange arrows represent a computational modeling workflow that fits a dynamic biophysical model to empirical BOLD time series in order to estimate model parameters and simulate underlying fast timescale neural activity.

Beyond differences in temporal resolution and sensitivity to time point ordering, methods for studying TVFC can be considered as taking one of two broad conceptual approaches to the challenge of studying brain dynamics. The first approach includes methods that attempt to estimate changes in FC (and/or identify FC states) directly from the observed BOLD data (e.g., sliding windows, Sakoglu et al., 2010; clustering, Calhoun & Adali, 2016; and HMMs, Vidaurre et al., 2017). The second approach includes methods that explicitly model the neural processes underlying changes in the observed BOLD data (e.g., simulations of the brain as a dynamical system, Breakspear, 2017; Park, Friston, Pae, Park, & Razi, 2018). These approaches are complementary, and we expect future work on BOLD TVFC to increasingly make use of these methods in combination. Below, we provide illustrative examples of each of the two approaches, but emphasize that these are not meant as a comprehensive review of all extant TVFC methods. Rather, they are intended to provide a general idea of the breadth of available methodological approaches. Figure 2 illustrates common workflows for TVFC analyses, while Table 2 provides a selection of key papers on BOLD TVFC, including a number of recent reviews of TVFC methods.

**Table 2.** Key papers on resting BOLD TVFC

**A method for evaluating dynamic functional network connectivity and task-modulation: Application to schizophrenia ** *Sakoglu et al., 2010;*https://doi.org/10.1007/s10334-010-0197-8 ** Time-frequency dynamics of resting-state brain connectivity measured with fMRI ** *Chang & Glover, 2010;*https://doi.org/10.1016/j.neuroimage.2009.12.011****Published almost simultaneously, these two papers were among the first to apply sliding-window and time-frequency analyses to the study of BOLD TVFC.

**Tracking whole-brain connectivity dynamics in the resting state** *Allen et al., 2014 (published online in 2012);*https://doi.org/10.1093/cercor/bhs352 One of the first papers to combine sliding-window analysis and clustering to estimate functional connectivity states and study their dynamics.

**Dynamic BOLD functional connectivity in humans and its electrophysiological correlates ** *Tagliazucchi et al., 2012;*https://doi.org/10.3389/fnhum.2012.00339 ** EEG correlates of time-varying BOLD functional connectivity ** *Chang et al., 2013;* https://doi.org/10.1016/j.neuroimage.2013.01.049 Two of the earliest studies to explore the electrophysiological basis of BOLD TVFC using simultaneous EEG/fMRI.

**Resting-state networks show dynamic functional connectivity in awake humans and anesthetized macaques** *Hutchison et al., 2013;*https://doi.org/10.1002/hbm.22058 One of the first studies to directly investigate the extent to which BOLD TVFC may exist independently of ongoing cognition.

**Dynamic functional connectivity: Promise, issues, and interpretations** *Hutchison et al., 2013*;https://doi.org/10.1016/j.neuroimage.2013.05.079 Important early review of BOLD TVFC findings and methods.

**Periods of rest in fMRI contain individual spontaneous events which are related to slowly fluctuating spontaneous activity** *Petridou et al., 2013;*https://doi.org/10.1002/hbm.21513 **Time-varying functional network information extracted from brief instances of spontaneous brain activity** *Liu and Duyn, 2013;*https://doi.org/10.1073/pnas.1216856110 Two early studies suggesting that BOLD FC may be shaped by the dynamics of transient coactivation patterns (CAPs).

**Time-resolved resting-state brain networks** *Zalesky et al., 2014;*https://doi.org/10.1073/pnas.1400181111 Early example of how sliding-window BOLD TVFC can be combined with graph theory analyses to investigate dynamic reorganization of functional brain networks during rest.

**Dynamic functional connectivity of the default mode network tracks daydreaming** *Kucyi and Davis, 2014;*https://doi.org/10.1016/j.neuroimage.2014.06.044 Early demonstration that resting BOLD TVFC is associated with time-resolved self-reports of ongoing cognition.

**The chronnectome: Time-varying connectivity networks as the next frontier in fMRI data discovery** *Calhoun et al., 2014*; https://doi.org/10.1016/j.neuron.2014.10.015 Review of BOLD TVFC methods, including an in-depth discussion of approaches that seek to estimate functional connectivity states.

**Evaluating dynamic bivariate correlations in resting-state fMRI: A comparison study and a new approach** *Lindquist et al., 2014;*https://doi.org/10.1016/j.neuroimage.2014.06.052 **Can sliding-window correlations reveal dynamic functional connectivity in resting-state fMRI?** *Hindriks et al., 2016;*https://doi.org/10.1016/j.neuroimage.2015.11.055 **On spurious and real fluctuations of dynamic functional connectivity during rest ** *Leonardi and Van De Ville, 2015;*https://doi.org/10.1016/j.neuroimage.2014.09.007 Three papers that carefully evaluate the potential pitfalls of sliding-window approaches and emphasize the importance of comparing against null models.

**Classification of schizophrenia and bipolar patients using static and dynamic resting-state fMRI brain connectivity** *Rashid et al., 2016;*https://doi.org/10.1016/j.neuroimage.2016.04.051 One of the first studies to demonstrate the superiority of BOLD TVFC over static FC for classifying individuals based on psychiatric diagnosis.
**The dynamic functional connectome: State-of-the-art and perspectives** *Preti et al., 2017;*https://doi.org/10.1016/j.neuroimage.2016.12.061 Detailed review of a wide range of methods for studying BOLD TVFC.

**Temporal metastates are associated with differential patterns of time-resolved connectivity, network topology, and attention** *Shine et al., 2016;*https://doi.org/10.1073/pnas.1604898113 A TVFC analysis of two large longitudinal single-subject datasets identified replicable temporal metastates with distinct functional network topologies, time-varying properties, and associations with cognition.

**On the stability of BOLD fMRI correlations** *Laumann et al., 2017;*https://doi.org/10.1093/cercor/bhw265 Influential paper challenging the notion that resting BOLD TVFC is related to ongoing cognition. Argues that resting BOLD is consistent with a stationary process and that resting TVFC can largely be explained by sampling variability, apparent head motion, and fluctuations in arousal.

**Interpreting temporal fluctuations in resting-state functional connectivity MRI** *Liegeois et al., 2017;*https://doi.org/10.1016/j.neuroimage.2017.09.012 Detailed exploration of which statistical properties are consistent with “dynamic” FC. Includes a detailed review of the concept of statistical stationarity, as well as an assessment of several common statistical models.

**Comparing test-retest reliability of dynamic functional connectivity methods** *Choe et al., 2017;*https://doi.org/10.1016/j.neuroimage.2017.07.005 **Replicability of time-varying connectivity patterns in large resting state fMRI samples** *Abrol et al., 2017;*https://doi.org/10.1016/j.neuroimage.2017.09.020 Two of the first large, systematic evaluations of the reliability of methods for estimating BOLD TVFC and identifying functional connectivity states.

**Brain network dynamics are hierarchically organized in time** *Vidaurre et al., 2017;*https://doi.org/10.1073/pnas.1705120114 HMM analysis reveals a rich hierarchical temporal structure in the pattern of transitions between FC states, and that individual differences in “meta state” occupancy are related to cognition.

**Dynamic models of large-scale brain activity** *Breakspear, 2017;*https://doi.org/10.1038/nn.4497 Accessible review of methods for modeling large-scale brain dynamics. Includes a primer on core concepts from dynamical systems theory.

**Neuronal origin of the temporal dynamics of spontaneous BOLD activity correlation** *Matsui et al., 2019;*https://doi.org/10.1093/cercor/bhy045 Simultaneous recording of calcium imaging and optical hemodynamics reveal a clear neural basis for BOLD TVFC, and that fluctuations in BOLD TVFC are related to transient neural CAPs.

**Simulations to benchmark time-varying connectivity methods for fMRI** *Thompson et al., 2018;*https://doi.org/10.1371/journal.pcbi.1006196 Recent work using multiple simulation strategies to undertake a systematic evaluation of the sensitivity of common TVFC methods. Provides an open-source toolbox for simulation and benchmarking.

**Putting the “dynamic” back into dynamic functional connectivity** *Heitmann and Breakspear, 2018;*https://doi.org/10.1162/netn_a_00041 Application of large-scale modeling to investigate which kinds of neural dynamics may give rise to BOLD TVFC. Argues that BOLD TVFC likely reflects complex nonlinear and nonstationary neural dynamics.

### Example 1: Data-Driven Methods for Estimating TVFC

One family of approaches for investigating time-varying functional connectivity focuses directly on the observed BOLD signal without explicitly modeling the underlying neural activity. These techniques typically approach the observed fMRI data as multivariate time series and seek to identify the time-resolved dependence structure between them. The most widely used approach in this class estimates pairwise correlations within a sliding window, resulting in time-resolved correlation matrices (one per window; Sakoglu et al., 2010). There are many variations on this theme, including the type of window used (square, Sakoglu et al., 2010; tapered, Allen et al., 2014; or exponentially decaying, Lindquist et al., 2014Lindquist, Xu, Nebel, & Caffo, 2014), the flexibility of the window (fixed, Allen et al., 2014; or adaptive, Lindquist et al., 2014; Yaesoubi et al., 2015), as well as the length of the window (Leonardi & Van De Ville, 2015; Liegeois et al., 2016; Sakoglu et al., 2010; V. M. Vergara, Mayer, Damaraju, & Calhoun, 2017; Zalesky & Breakspear, 2015). Other (windowless) methods estimate FC without assuming locality of the neighboring time points (Yaesoubi et al., 2018), or utilize time-frequency methods to estimate instantaneous FC using phase synchrony (Chang & Glover, 2010; Pedersen, Omidvarnia, Zalesky, & Jackson, 2018; Yaesoubi et al., 2015). Regardless of the particular method used, a common next step is to assess the potential time-varying properties of the resulting time-resolved FC estimates, and to explore possible associations with other dynamic phenomena (e.g., behavioral performance, Kucyi, Esterman, Riley, & Valera, 2016; Patanaik et al., 2018; or cognitive state, Kucyi & Davis, 2014). TVFC estimates can also be summarized through the use of descriptive statistics (e.g., variance, Chang & Glover, 2010; Kucyi, Salomons, & Davis, 2013) or methods that attempt to identify functional connectivity “states.” Methods for identifying states include sliding windows + clustering (e.g., Allen et al., 2014), hidden Markov models (HMMs; Shappell, Caffo, Pekar, & Lindquist, 2019; Vidaurre et al., 2017), change-point modeling (Cribben, Haraldsdottir, Atlas, Wager, & Lindquist, 2012; Xu & Lindquist, 2015), and windowless dynamic connectivity (Yaesoubi et al., 2018). After identifying states, it is possible to estimate a variety of parameters such as mean dwell times, transition probabilities, and graph theoretic measures that describe the observed FC patterns and brain dynamics (e.g., network modularity; Zalesky et al., 2014). These parameters can then be probed for association with measures of inter- or intraindividual differences (e.g., Beaty et al., 2018; Marusak et al., 2018; Vidaurre et al., 2017). State-based approaches can differ in whether they assume smooth transitions between states (Allen et al., 2014; Ou et al., 2015) or instantaneous reconfigurations (Liu, Zhang, et al., 2018; Yaesoubi et al., 2018), their focus on a particular signal domain (e.g., frequency, Yaesoubi et al., 2015; time, Allen et al., 2014; or space, S. Ma, Calhoun, Phlypo, & Adali, 2014), and whether the state definitions are “hard” or “soft” (i.e., whether each time point exhibits a single state, Allen et al., 2014; or is composed of a mixture of multiple states, Leonardi, Shirer, Greicius, & Van De Ville, 2014; R. L. Miller et al., 2016). Temporal network theory, a subfield of graph theory, can also be used to quantify how functional network properties change over time (Holme & Saramäki, 2012; W. H. Thompson, Brantefors, & Fransson, 2017; Yu et al., 2015). In all cases, it is critical to benchmark these statistics (i.e., the TVFC estimates or state-related parameters) against those derived from reference data that embody a null or alternative hypothesis (e.g., that FC is “static” and does not in fact vary over time). We return to the issue of null models in the section on statistical challenges in studying BOLD TVFC, below.

### Example 2: Modeling the Underlying Neuronal Dynamics

In contrast to methods that seek to analyze the observed BOLD signal directly, a second family of approaches instead aims to model the underlying neural fluctuations and interactions that give rise to BOLD TVFC. This approach posits that observed BOLD time series are generated by underlying nonlinear brain dynamics that are then corrupted by measurement noise. Under this view, activity in large-scale neural systems is inherently dynamic and exhibits complex phenomena such as partial synchronization, multistable attractor landscapes, and edge-of-chaos behavior indicative of criticality (Cocchi, Gollo, Zalesky, & Breakspear, 2017; Deco, Jirsa, Robinson, Breakspear, & Friston, 2008; Heitmann & Breakspear, 2018; Roberts, Boonstra, & Breakspear, 2015; Zalesky et al., 2014). These dynamics generate physiological time series with highly nonlinear structure and can be formally modeled by biophysically derived differential equations. By combining these equations with models of the observation process (e.g., neurovascular coupling), it is possible to simulate how these underlying dynamics would manifest in the BOLD signal (i.e., after the addition of measurement noise). There are a wide variety of multiscale models of interconnected pools of neurons, including neural mass and neural field models (Bojak, Oostendorp, Reid, & Kotter, 2010; Breakspear, 2017; Deco et al., 2008). These have been shown to produce neurobiologically plausible behaviors such as generalized synchronization, metastability, and multistability (Breakspear, 2017; Deco et al., 2008; Golos, Jirsa, & Dauce, 2015; Heitmann & Breakspear, 2018; Roberts et al., 2019). Exploratory computational work involves adjusting the model structure and tuning parameters in order to obtain, through simulation, synthetic BOLD data that exhibits similar dependence structure and dynamics to empirical observations (e.g., Deco, Cruzat, & Kringelbach, 2019; Demirtas et al., 2019; Kashyap & Keilholz, 2019; P. Wang et al., 2019). Model-based approaches need to make strong assumptions about the processes that generate observed BOLD data (Deco et al., 2008). Under these assumptions, it is possible to estimate from observed BOLD data the parameters of these models, and thus the underlying neural dynamics (including time-varying aspects; e.g., Deco et al., 2019; Kashyap & Keilholz, 2019). This process is known as model inversion. Models can be evaluated using a variety of methods (e.g., information criteria) that consider how well they fit observed data while penalizing model complexity. Careful model construction facilitates the testing of specific hypotheses about underlying dynamics, as well as validation of findings from approaches that model the BOLD signal directly (Zalesky et al., 2014).

### A Rich Diversity of Methods for Studying TVFC

There is no single “best” method for studying time-varying functional connectivity; the choice of analytic strategy should be informed by the available data and the particular questions under investigation. Different approaches provide different (complementary) perspectives on the data, and a full understanding of the factors giving rise to TVFC and their relationship to cognition and behavior will likely necessitate integrating knowledge gained through the application of a wide variety of methods (see Box 3). Some approaches (e.g., Example 1) make minimal (or no) explicit assumptions about the underlying biology, while others (e.g., Example 2) seek to model the biophysical parameters directly. Improved biological specificity is often accompanied by greater model complexity and more extensive explicit model assumptions. That said, methods that directly model the observed BOLD signal can also be highly statistically articulated (e.g., HMMs) and come with their own assumptions (e.g., that the data are best represented by a limited number of states) that are often just as strong as assumptions made by biophysical models.

Highly articulated “data-driven” models (e.g., autoregressive models, Rogers, Katwal, Morgan, Asplund, & Gore, 2010; or HMMs, Vidaurre, Abeysuriya, et al., 2018) may explain the data very well without recourse to biological assumptions, but do not provide information about the underlying neuronal dynamics without additional parameterization. As we learn more about brain physiology and dynamics, additional biologically informed constraints can be added to restrict the space of possible model solutions and improve the ability of these methods to accurately describe the neural processes underlying noisy BOLD data. In contrast, dynamical (nonlinear) systems theory provides an adequately rich parameterization to enable explicit exploration of how networks of neurons—modeled as coupled oscillators or populations of spiking neurons—may give rise to the observed BOLD signal. Scientific investigation of TVFC is likely to be enriched by the application of both approaches, as they have complementary strengths, and the results from one perspective can inform the application of the other. For example, data-driven models of the observed BOLD signal can yield new biological hypotheses, that, if confirmed, can then be integrated into richer empirically grounded dynamical models. Two recent studies on how anatomical features shape static FC provide an excellent example of how empirical work can inform modeling efforts, and vice versa. P. Wang et al. (2019) inferred a hierarchy of recurrent anatomical connectivity across cortical regions by inverting a large-scale dynamic circuit model fit to empirically observed resting fMRI data. Complementary work by Demirtas et al. (2019) used MRI to map anatomical hierarchy as indexed by cortical myelination, and found that incorporating hierarchy information into a biophysical model of neural dynamics significantly improved the fit to human rfMRI data. Taken together, these studies suggest that connectional hierarchy plays a fundamental role in shaping intrinsic neural dynamics. We expect that future work incorporating characteristics of empirically observed TVFC into dynamical models will provide similarly important insights into brain organization and function.

Box 3. The elusive concept of dynamic functional connectivity The term “dynamic functional connectivity” has been used to refer to a wide range of approaches for studying time-varying aspects of brain function. These approaches differ in the insights they offer into brain dynamics, and it is important to distinguish which inferences can (and cannot) be drawn from each method. Below, we briefly outline how four broad classes of TVFC methods can be used to expand our understanding of brain function.

**Time-resolved estimates of functional connectivity:** Empirical estimates of time-resolved functional connectivity allow scientists to explore how the strength of interregional coupling varies over time. These estimates form the basis of empirical studies of TVFC. In their most basic form (i.e., time-resolved correlations), they can provide insight into the trajectories by which static (“time-averaged”) FC is realized. Time-resolved estimates also allow for fine-grained evaluations of the relationship between FC and ongoing cognition, as well as how summary measures (e.g., variability of FC) may be related to phenotypic traits in health and disease.

**Models of states and transitions:** Many empirical studies of TVFC also seek to estimate transient “brain states” and their transitions. In this paradigm, each state describes a different pattern of whole-brain activity or functional connectivity. Different models impose varying constraints on the estimated states, such as whether they manifest in isolation (one state per time point) or in combination (a mix of states at each time point). The dynamics of these states (e.g., time spent in each state, the probability of transitioning between states) can provide a detailed portrait of how functional relationships reorganize through time. Formal model selection and comparison (e.g., using information-theoretic criteria) allows for the evaluation of which models best describe the observed data, and thus permit adjudication of competing hypotheses about data-generating processes.

**Comparison to surrogate (null) data:** Insight into the dynamical properties of a system can also be achieved by comparing observed data to surrogate data that lack a particular statistical feature of interest. For example, one can generate surrogate “null” time series that have the same low-order features as empirical data (e.g., mean, variance, spatiotemporal correlation structure) but lack a higher order feature proposed to exist in the real data (e.g., switching dynamics). The strength of this approach is that it draws from a rich existing literature on time series analysis and enables testing of specific hypotheses about the dynamical properties of an observed time series. Care must be taken to ensure that the tests undertaken are sufficiently narrow and are interpreted as such. For example, claims should be made about the presence or absence of a particular statistical feature rather than “dynamic” FC in general, as “dynamic” phenomena can exist under a wide range of conditions.

**Modeling of nonlinear brain dynamics:**Unlike the three approaches above that begin with empirically measured BOLD data, it is also possible to instead begin the study of TVFC by constructing a detailed biophysical model of the underlying processes thought to give rise to TVFC. With appropriate model fitting and tuning, it is possible to invert the observed data into a generative model, and then study the complex (fast timescale) dynamical properties of that model that would normally be obscured by the measurement process. Having established a model of the dynamical processes underlying the observed data, researchers can undertake detailed mechanistic investigations of complex neural dynamics and their relationship to BOLD TVFC.

## STATISTICAL CHALLENGES IN STUDYING BOLD TVFC

Before diving into questions about the biological basis and cognitive relevance of resting BOLD TVFC, we must first ask whether there is statistical evidence for this phenomenon: Does functional connectivity estimated from resting BOLD fMRI actually vary over time? In this section, we discuss the importance of testing TVFC estimates against null models, review the role of sampling variabilityin TVFC estimation, and describe approaches for evaluating and validating TVFC methods.

### The Importance of Testing Against Null Models

Any method designed to estimate TVFC will inevitably return time-resolved estimates of functional connectivity that vary to some degree with time (Lindquist et al., 2014). Researchers must therefore carefully evaluate whether the observed TVFC estimates significantly deviate from those that might have been obtained from time series generated by a process that lacks a particular property of interest (e.g., state switching, fluctuating FC). It is then possible to compare empirically observed time series with a suitable surrogate “null” distribution, typically generated through simulation or nonparametric resampling (Breakspear, Brammer, Bullmore, Das, & Williams, 2004; Prichard & Theiler, 1994). Multiple methods have been developed to generate surrogate data, including methods that represent a null model based on a specific system (Hindriks et al., 2016), biophysical models that simulate different classes of dynamics in the brain (Heitmann & Breakspear, 2018), and techniques that are designed to test the properties of specific methods used to estimate TVFC (Allen et al., 2014; Shakil, Lee, & Keilholz, 2016).

When evaluating TVFC through comparison with null models, it is important to carefully consider both the features of the process used to generate null data, as well as the test statistic used to evaluate whether observed TVFC estimates deviate from that null. For example, although some work has focused on statistical stationarity as a feature of interest (Laumann et al., 2017), subsequent work (Liegeois et al., 2017; R. L. Miller et al., 2018) has demonstrated that the space of stationary models includes many processes that exhibit TVFC (e.g., HMMs with switching covariance structure). Thus, statistical stationarity is not necessarily tantamount to static functional connectivity. Conversely, evidence of nonstationarity does not always imply the presence of a “meaningful” change and/or trend in the data (Koutsoyiannis, 2011; Lins, 2012). Likewise, it is important to keep in mind that TVFC estimates that fail to differ significantly from a given null do not necessarily equate to “meaningless fluctuations.” Rather, such fluctuations could be consistent with a more restricted space of stationary stochastic models that may still have scientifically interesting properties (i.e., have heavy spatial and temporal tails; Cocchi et al., 2017; R. L. Miller et al., 2018; Roberts et al., 2015).

It remains an open question which time series features and null models are most appropriate for evaluating various aspects of TVFC, and as such we refrain from making any specific recommendations. That said, the case of statistical stationarity provides a good example of the process by which one might assess the properties of null models and time series features, test for the presence of candidate features in empirical data, and interpret the results of these analyses. Laumann et al. (2017) proposed testing for the presence of TVFC by evaluating the multivariate kurtosis of rfMRI time series, with multivariate kurtosis used as a test statistic to assess the stationarity of the time series, and stationarity used as an index of the extent to which the time series exhibit “dynamic” fluctuations in FC. The values of multivariate kurtosis observed by Laumann et al. were insufficient to reject the null of a stationary process, and the authors interpreted this finding as evidence against the presence of TVFC in rfMRI. However, as mentioned above, subsequent analyses by Liegeois et al. (2017) found that multiple commonly used “dynamic” models (e.g., HMMs, autoregressive models) are statistically stationary, and that this stationarity exists even for models with switching covariance structure (HMMs). Additional work by R. L. Miller et al. (2018) found that time series properties leading to elevated multivariate kurtosis (which Laumann et al. interpreted as evidence of nonstationarity) are sometimes more consistent with stationary than nonstationary processes. Taken together, these results suggest that (a) stationary processes are consistent with the presence of TVFC, and (b) multivariate kurtosis is likely a poor proxy for statistical stationarity. More generally, the papers by Laumann, Liegois, and Miller provide an excellent example of how the research community can work together to begin establishing a consensus on which time series properties and null models are most appropriate for testing various aspects of TVFC.

### The Role of Sampling Variability

Sampling variability is a key consideration for statistical inference. BOLD FC is typically estimated as the bivariate correlation between two time series, and a peculiar property of correlations of time series (first discussed over 90 years ago; Bartlett, 1935) is that one can obtain high correlation coefficients even in the absence of a real relationship. This phenomenon (resulting from autocorrelation) can largely be summarized as an issue of sampling variability, which refers to how much a statistic varies across realizations of the data. The lower the sampling variability, the more precise the subsequent inference (e.g., confidence intervals and hypothesis tests).

As an example, consider the sampling variability of the sliding-window approach. Because sliding windows (and other TVFC methods) estimate a series of correlations, it can be useful to think of these values as “repeated samples” of correlations across time. From this perspective, the key question being asked when evaluating TVFC estimates is whether each sample was drawn from the same distribution (static FC) or from distinct distributions (TVFC). If we choose a small window size, the correlation coefficient will be based on few data points; this gives rise to larger sampling variability. Thus, short window lengths may give rise to signals that show compellingly “dynamic” changes in correlation across time, even if the FC is actually static (Hlinka & Hadrava, 2015; Leonardi & Van De Ville, 2015; Lindquist et al., 2014). This problem becomes less pronounced as window length increases, but longer windows come at the cost of reduced sensitivity to transient changes in correlation. In addition, if overlapping windows are used, an autocorrelation (beyond that already present because of the smoothness of the BOLD signal) is induced in the estimated TVFC values, which can make changes in FC appear artificially smooth (Lindquist et al., 2014). That said, recent work (V. Vergara, Abrol, & Calhoun, in press; V. M. Vergara et al., 2017) suggests that the optimal window length to minimize these concerns may be shorter than the minimum of ∼60 s that has been previously recommended (Leonardi & Van De Ville, 2015; Zalesky & Breakspear, 2015), and one can consider the choice of window size to be a tunable filter that can be optimized based on the question of interest (Lindquist et al., 2014; V. Vergara et al., in press).

### Establishing the Sensitivity and Reliability of TVFC Methods

Prior to the use of any new method, it is crucial to systematically evaluate the accuracy and reliability of its performance. One key metric of algorithmic accuracy is sensitivity, which for TVFC methods is the ability to accurately recover TVFC from noisy data. As the “ground truth” of the fluctuating neural interactions underlying TVFC is often unknowable (and perhaps even undefined), evaluations of sensitivity typically make use of simulated data containing a known TVFC signal of interest (i.e., a particular pattern of time-varying dependence structure). A variety of simulation tools are available to help researchers evaluate how TVFC methods perform under a range of different data-generating conditions (Erhardt, Allen, Wei, Eichele, & Calhoun, 2012; Sanz Leon et al., 2013; W. H. Thompson et al., 2018; Welvaert & Rosseel, 2014). Results from sensitivity analyses suggest not only that different TVFC methods have different degrees of sensitivity, but that sensitivity is influenced by factors such as window length and data quantity (i.e., scan duration; Hindriks et al., 2016; W. H. Thompson et al., 2018).

It is also critical to demonstrate that estimates of BOLD TVFC are reliable enough to serve as robust markers of ongoing cognition and/or individual differences. Recent work has shown that whole-brain patterns of TVFC at rest are largely reproducible across individuals (Abrol et al., 2017; Choe et al., 2017; Vidaurre, Abeysuriya, et al., 2018), even when considering data from multiple scan sites and heterogeneous populations (Abrol et al., 2017). Complementary work has shown that individual differences in resting TVFC dynamics show good test-retest reliability (Choe et al., 2017; Liao, Cao, Xia, & He, 2017; Vidaurre, Abeysuriya, et al., 2018). These studies satisfy an important prerequisite for continued research into resting TVFC, and future work should continue to refine our understanding of which factors influence the reliability of these measures (Lehmann, White, Henson, Cam, & Geerligs, 2017). Additional work is also necessary to assess which properties of TVFC are stable over time within an individual (i.e., “trait” characteristics; Geerligs, Rubinov, Cam, & Henson, 2015) and which are modulated by the particular experimental context or cognitive state.

## THE BIOLOGICAL BASIS OF BOLD TVFC

### Cross-Modal Comparisons of BOLD TVFC and Direct Measures of Neural Activity

FMRI is unique in its ability to noninvasively measure and localize activity simultaneously across the entire brain at relatively high spatial resolution. This has made it the modality of choice for many researchers interested in understanding large-scale brain dynamics (especially in humans). However, the BOLD signal is a noisy, indirect measure of underlying neural activity, and the sluggish hemodynamic response places a fundamental limit on the temporal resolution of TVFC estimated from fMRI data. It is well established that the shape of the hemodynamic response function varies across brain areas (Handwerker, Ollinger, & D’Esposito, 2004) and individuals (Aguirre, Zarahn, & D’Esposito, 1998), and emerging work suggests that neurovascular coupling may also vary across behavioral and bodily states (Elbau et al., 2018; Lecrux & Hamel, 2016; Winder, Echagarruga, Zhang, & Drew, 2017). While these and other factors can complicate the neurophysiological interpretation of fMRI findings, we do not believe they preclude the use of BOLD fMRI for studies of time-varying neural interactions. Rather, they strongly motivate the need to validate and extend findings from fMRI through comparison with other modalities.

If we wish to use BOLD TVFC to study temporal fluctuations in interregional neural interactions, it is first necessary to establish a firm neural basis for regional BOLD activity and functional connectivity. Intracranial recordings have consistently revealed a positive correlation between the regional BOLD signal and electrophysiological high-frequency broadband power (∼ 50 − 150 Hz, also sometimes referred to as “high gamma”; Logothetis, Pauls, Augath, Trinath, & Oeltermann, 2001; K. J. Miller, Weaver, & Ojemann, 2009; Mukamel et al., 2005; Nir et al., 2007; Scholvinck, Maier, Ye, Duyn, & Leopold, 2010), and patterns of FC estimated from fluctuations in high-frequency broadband power reliably exhibit similar topography to intrinsic BOLD FC networks when the two modalities are compared within the same individuals (Foster, Rangarajan, Shirer, & Parvizi, 2015; Hacker, Snyder, Pahwa, Corbetta, & Leuthardt, 2017; He, Snyder, Zempel, Smyth, & Raichle, 2008; Kucyi, Schrouff, et al., 2018). Studies have also observed correspondence between patterns of BOLD FC and interareal correlations in the band-limited power of a range of lower frequencies (e.g., delta, theta, alpha, and beta), which can be detected using both invasive electrophysiology (Foster et al., 2015; Hacker et al., 2017; Lu et al., 2007; L. Wang, Saalmann, Pinsk, Arcaro, & Kastner, 2012) and MEG (Baker et al., 2014; Brookes et al., 2011; Hipp, Hawellek, Corbetta, Siegel, & Engel, 2012; Hipp & Siegel, 2015; Houck et al., 2017). The correspondence observed between BOLD FC and electrophysiological FC at these lower frequencies may be specific to particular functional brain networks (Hacker et al., 2017; Hipp & Siegel, 2015).

Recently, multimodal recording approaches have been adopted to directly investigate the neurophysiological basis of BOLD TVFC (see G. J. Thompson, 2018, for a review). These studies suggest that like static FC, BOLD TVFC may reflect fluctuations of electrophysiological FC across multiple frequency bands. Simultaneous fMRI and intracranial recordings in rats found that BOLD TVFC between left and right somatosensory areas tracked changes in FC calculated from band-limited electrophysiological power, and that these associations exist across several canonical frequency bands (theta, beta, and gamma; G. J. Thompson, Merritt, et al., 2013). Preliminary support for these relationships in humans comes from TVFC analyses of simultaneously recorded EEG-fMRI data, which have found associations between BOLD TVFC and changes in power across multiple frequency bands (delta, theta, alpha, beta, and low-gamma; Allen, Damaraju, Eichele, Wu, & Calhoun, 2018; Chang et al., 2013; Tagliazucchi et al., 2012). Unfortunately, because of the poor spatial resolution of EEG, these studies are unable to speak directly to the electrophysiological basis of spatially specific variations in coupled activity between brain regions. However, studies using MEG (which provides improved spatial localization relative to EEG for many cortical regions) have observed time-varying interregional correlations of band-limited power that have similar spatial topography to BOLD FC networks (de Pasquale et al., 2010; Vidaurre, Hunt, et al., 2018). The temporal correspondence of these effects with BOLD TVFC remains uncertain, as MEG cannot be recorded simultaneously with fMRI.

Studies have shown that fluctuations in local field potentials at low frequencies directly comparable to BOLD fluctuations (<1 Hz) also contribute substantially to measures of functional connectivity and TVFC (Grooms et al., 2017; He et al., 2008; Hiltunen et al., 2014; Pan, Thompson, Magnuson, Jaeger, & Keilholz, 2013; G. J. Thompson, Pan, Magnuson, Jaeger, & Keilholz, 2014). Notably, these infraslow fluctuations in neural activity have been linked to quasiperiodic spatiotemporal patterns of BOLD fluctuations that involve coordinated propagation of activity across the brain (Grooms et al., 2017; G. J. Thompson, Pan, Magnuson, et al., 2014). In this vein, studies have successfully modeled BOLD TVFC as being driven by transient periods of coordinated high-amplitude coactivations (Karahanoglu & Van De Ville, 2015; Tagliazucchi, Siniatchkin, Laufs, & Chialvo, 2016). These coactivation patterns are reliable across individuals (Gutierrez-Barragan, Basson, Panzeri, & Gozzi, 2019; Liu & Duyn, 2013) and may preferentially occur at distinct phases of the infraslow global signal (Gutierrez-Barragan et al., 2019). In addition to observing a close correspondence between windowed TVFC calculated from optically imaged hemodynamic signals and simultaneously recorded calcium transients, recent work in rodents found that variation in transient neural coactivations was associated with fluctuations in TVFC, and that neither calcium nor hemodynamic TVFC were consistent with simulations that assumed stationary covariance structure (Matsui et al., 2019).

Overall, it seems possible (even probable) that multiple, potentially dissociable neurophysiological processes simultaneously contribute to time-varying BOLD activity and functional connectivity, and there is good reason to believe that the heterogeneity of electrophysiological frequency bands reported as being associated with BOLD static FC and TVFC is not merely artifactual or due to experimental variability. Different bands of electrophysiological activity likely reflect distinct neurophysiological processes (Buzsaki & Draguhn, 2004), and recent work with human intracranial recordings—including within prominent nodes of canonical networks (e.g., default, dorsal attention)—has revealed that different frequency ranges (e.g., high-frequency broadband versus alpha) of resting TVFC within a network often temporally diverge from one another (Kucyi, Schrouff, et al., 2018). Indeed, it has been suggested that there may be an “inverse problem” for BOLD functional connectivity in that there could be different underlying contributors at different moments (Leopold & Maier, 2012). While this certainly complicates the neurophysiological interpretation of studies of BOLD FC, recent work has begun to pull apart how distinct aspects of neural signaling may underlie different aspects of BOLD activity and functional connectivity. The results of these studies suggest that while infraslow oscillations and fluctuations in the band-limited power of higher frequencies of local field potential are both associated with fluctuations in the BOLD signal, they may reflect different underlying neurophysiological phenomena (G. J. Thompson, Pan, Billings, et al., 2014), and that quasi-periodic patterns of BOLD activity may have different neural correlates than windowed TVFC (G. J. Thompson, Pan, Magnuson, et al., 2014).

### Neuromodulatory Influences on BOLD TVFC

The effect of neuromodulators on neural activity and connectivity (both functional connectivity and synaptic strength) is a critical but often overlooked aspect of brain function. Regional and global release of these molecules can lead to drastic changes in the dynamics of neural circuits (Bargmann, 2012; Bargmann & Marder, 2013), and growing evidence suggests that neuromodulatory systems play a key role in triggering and sculpting the reconfiguration of functional brain networks across diverse behavioral states (Alavash et al., 2018; Guedj, Meunier, Meunier, & Hadj-Bouziane, 2017; Guedj, Monfardini, et al., 2017; Hermans et al., 2011; Shine, Aburn, Breakspear, & Poldrack, 2018; Shine et al., 2019; van den Brink, Pfeffer, & Donner, 2019; van den Brink et al., 2016; Zerbi et al., 2019). In line with these findings, experimental manipulation of the neuromodulatory neurotransmitters dopamine (e.g., Shafiei et al., 2018), noradrenaline (e.g., Shine, van den Brink, Hernaus, Nieuwenhuis, & Poldrack, 2018), acetylcholine, and serotonin (e.g., Klaassens et al., 2017) have all been associated with time-varying alterations of BOLD FC. These studies typically involve a placebo-controlled design in which prior to scanning, subjects either ingest a pharmacological agent (e.g., a neurotransmitter agonist, antagonist, or reuptake inhibitor) or are administered a diet depleted in particular essential amino acids (e.g., tyrosine and phenylalanine) such that the stockpiles of neurotransmitters reliant on these chemicals for synthesis become exhausted. Given that these manipulations target endogenous neuromodulatory systems, it is reasonable to suspect that intrinsic and task-related fluctuations in neuromodulatory signaling may be a core mechanism underlying TVFC (Shine, 2019; van den Brink et al., 2019). In addition to their diverse and potent effects on neural activity, neuromodulators can also influence neurovascular coupling (Bruinsma et al., 2018; Lecrux & Hamel, 2016). As such, care must be taken to ensure that the effects observed in fMRI studies of pharmacological manipulation are indeed related to changes in neural activity (e.g., Shine, van den Brink, et al., 2018), and not simply due to altered hemodynamics.

### The Role of Arousal and Sleep State in Driving Fluctuations in TVFC

Arousal is an important dimension of brain function to consider when analyzing large-scale neuronal activity (Munk, Roelfsema, Konig, Engel, & Singer, 1996; D. Pfaff, Ribeiro, Matthews, & Kow, 2008; Steriade, McCormick, & Sejnowski, 1993), especially when seeking to relate ongoing neural fluctuations to cognition and behavior (Mather, Clewett, Sakaki, & Harley, 2016; Sara & Bouret, 2012; van Swinderen, Nitz, & Greenspan, 2004). It has been known for some time that rfMRI functional connectivity patterns change when subjects fall asleep (Duyn, 2011; Horovitz et al., 2008; Larson-Prior et al., 2009), even for very short periods of time (just a few seconds, i.e., “microsleeps”; C. Wang, Ong, Patanaik, Zhou, & Chee, 2016). Resting BOLD FC networks observed during sleep exhibit diminished temporal autocorrelation (Tagliazucchi et al., 2013), and estimates of TVFC are sensitive to fluctuations in drowsiness, arousal, and vigilance (Damaraju, Tagliazucchi, Laufs, & Calhoun, 2018; Haimovici, Tagliazucchi, Balenzuela, & Laufs, 2017; Laumann et al., 2017; Patanaik et al., 2018; Tagliazucchi & Laufs, 2014). Similarly, simultaneous EEG-fMRI studies have shown that EEG patterns known to occur during sleep correspond to distinct aspects of BOLD TVFC fluctuations (Allen et al., 2018; Chang et al., 2013; Damaraju, Hjelm, Plis, & Calhoun, 2017; Tagliazucchi & Laufs, 2014), and some studies suggest that fluctuations in arousal may explain a large proportion of variance in TVFC (Chang et al., 2013; Laumann et al., 2017; Tagliazucchi & Laufs, 2014). This may pose a particular problem when comparing groups or individuals with differing levels of drowsiness (e.g., Parkinson’s disease; Knie, Mitra, Logishetty, & Chaudhuri, 2011), and motivates the need to include sleep assessments and measurements of arousal in studies of static FC and TVFC.

However, despite concerns raised by the association between level of arousal and BOLD TVFC, it is important to note that sleep and level of arousal (as well as other global neuronal processes) often relate to cognition in nontrivial ways (Mather et al., 2016; Sara & Bouret, 2012; Walker, 2009). Changes in level of arousal are not purely binary (sleep vs. wake), but rather exist along a continuum. Subtle changes in arousal (e.g., epochs of heightened awareness/focus vs. high distractibility) provide important constraints on cognitive processing (e.g., the vigilance required to respond accurately during continuous performance; M. Rosenberg, Noonan, DeGutis, & Esterman, 2013; or stimulus detection tasks; Sadaghiani & D’Esposito, 2015), while sleep deprivation and drowsiness can have a major negative impact on behavioral performance (Gillberg, Kecklund, & Akerstedt, 1994; Harrison & Horne, 2000). Given this close relationship between cognition and arousal, and the fact that fluctuations in arousal are largely driven by the activity of ascending brainstem neuromodulatory systems (Liu, de Zwart, et al., 2018; D. Pfaff et al., 2008; D. W. Pfaff, Martin, & Faber, 2012), it is perhaps to be expected that if TVFC relates to cognition then it should also correlate at least moderately with arousal. Taking this perspective questions whether cognition and arousal effects on TVFC can ever be adequately disentangled in a way that does not “throw out the baby with the bath water.” Further, disambiguating the neural and physiological correlates of arousal from their consequences (e.g., changes in head motion, heart rate, and respiration) is not straightforward, and thus caution should be taken in treating them as artifactual.

Beyond wakeful states, information processing and homeostatic processes that occur during sleep play a critical role in memory consolidation (Genzel, Kroes, Dresler, & Battaglia, 2014; McKenzie & Eichenbaum, 2011; Walker, 2009), and have been implicated in a wide range of cognitive processes including creativity and emotion regulation (Walker, 2009). As such, while TVFC observed during sleep likely has little or no *immediate* cognitive or behavioral relevance, it may very well reflect important information processing that can impact subsequent waking thought and action. Such a perspective is consistent with the notion that TVFC likely reflects a variety of conscious and unconscious cognitive processes, as well as intrinsic noncognitive processes (Kucyi, 2018). Determining which (if any) aspects of BOLD TVFC are sensitive to these “off-line” cognitive processes—as well as whether they can be distinguished from purely physiological homeostatic processes—will be a key challenge for our field.

## COGNITIVE AND BEHAVIORAL RELEVANCE OF RESTING BOLD TVFC

A key attraction of BOLD TVFC is the potential that it may be used to study the neural basis of cognition and behavior, which are inherently dynamic. There is growing consensus that fMRI is sensitive to changes in functional connectivity that accompany switches between externally cued tasks, and there is considerable interest in extending this work to studies of cognition during the “resting” state. While the unconstrained nature of the resting paradigm presents important challenges, it also provides exciting opportunities. Here, we review the evidence both for and against the cognitive relevance of TVFC, with an emphasis on cognition during rest.

### Evidence That Time-Varying Functional Connectivity Is Related to Ongoing Cognition and Behavior

##### BOLD TVFC tracks cognitive task performance.

There is robust evidence that static BOLD FC patterns flexibly reconfigure across a range of cognitive and behavioral states (Cohen & D’Esposito, 2016; Cole, Bassett, Power, Braver, & Petersen, 2014; Cole et al., 2013; Gratton et al., 2018; Mattar, Cole, Thompson-Schill, & Bassett, 2015; Shirer, Ryali, Rykhlevskaia, Menon, & Greicius, 2012Shirer, Ryali, Rykhlevskaia, Menon, & Greicius, 2012; see Shine & Poldrack, 2018, for a recent review of this literature). The most well-studied examples involve the modulation of FC during cued task performance. In typical experiments, subjects are presented with one or more externally cued cognitive tasks and estimates are made of static FC during task performance. These studies are complemented by a growing literature on task-related TVFC, which suggests that BOLD TVFC measures are sensitive to fluctuations in short-timescale changes in FC both within and across a wide range of behavioral tasks (for recent reviews, see Cohen, 2018; Gonzalez-Castillo & Bandettini, 2018). TVFC can be used to predict which of multiple tasks an individual is engaged in (Gonzalez-Castillo et al., 2019; Gonzalez-Castillo et al., 2015; Xie, Zheng, et al., 2019), and differences in TVFC during task execution have been associated with both subjective emotional experience (Tobia, Hayashi, Ballard, Gotlib, & Waugh, 2017) and objective measures of task performance (Shappell et al., 2019; Shine, Bissett, et al., 2016; Xie, Gonzalez-Castillo, et al., 2019). TVFC methods have also been used to identify functional network configurations associated with different task epochs (Soreq, Leech, & Hampshire, 2019), condition-specific pretrial preparatory processes (Ekman, Derrfuss, Tittgemeyer, & Fiebach, 2012), and subsequent behavioral performance (Ekman et al., 2012; Sadaghiani, Poline, Kleinschmidt, & D’Esposito, 2015). Together, these studies provide compelling evidence that the functional macroscale architecture of the brain as measured with BOLD FC is sensitive to the dynamics of cognitive and behavioral states.

Prior knowledge of stimulus timing and the ability to tie observed TVFC to objectively measured fluctuations in behavior make task-based studies a highly interpretable validation of the cognitive relevance of BOLD TVFC. However, this approach is not without criticism. It is possible that the TVFC patterns associated with these tasks are altered by the somewhat artificial temporal, serial, and repetitive nature of most behavioral paradigms used in cognitive neuroscience. This problem may potentially be mitigated by using tasks with greater ecological validity (e.g., naturalistic viewing or listening; see below). In addition, task-related changes in estimates of FC can be driven by relatively trivial changes in coordinated activity, such as those resulting from stimulus-induced coactivation (Cole et al., 2019; Duff, Makin, Cottaar, Smith, & Woolrich, 2018). Several approaches exist that attempt to address this concern, including regression of stimulus-related effects (also known as “background connectivity”; Norman-Haignere, McCarthy, Chun, & Turk-Browne, 2012) and psychophysiological interaction models (PPI; O’Reilly, Woolrich, Behrens, Smith, & Johansen-Berg, 2012). However, if the task model is at all misspecified (e.g., the use of an incorrect hemodynamic response function, failure to model all relevant aspects of the task), then apparent correlations could still be driven by common stimulus-evoked activity rather than by interregional neural interactions per se (Cole et al., 2019).

##### Stimulus-related cognitive dynamics and “pseudo-rest” paradigms.

Paradigms with minimal explicit task demands but known time-varying stimulus properties may be particularly useful for establishing the immediate cognitive relevance of TVFC in the absence of explicitly cued task switches. A growing number of experiments using “naturalistic stimuli” (e.g., free viewing or listening to movies or audio recordings; Sonkusare, Breakspear, & Guo, 2019) provide evidence that time-varying properties of the BOLD signal track fluctuations in stimulus-related cognitive state (Hasson, Nir, Levy, Fuhrmann, & Malach, 2004; Hejnar, Kiehl, & Calhoun, 2007; Lerner, Honey, Silbert, & Hasson, 2011; Nguyen et al., 2017; Regev et al., 2019). This type of experiment allows researchers to study correlated activity *across individuals* to identify brain areas whose fluctuations are temporally locked to features of a continuous complex stimulus (intersubject correlation; Hasson et al., 2004). Because coherent intersubject fluctuations (beyond perhaps those due to study-induced fatigue) are unlikely to occur during otherwise unconstrained listening/viewing, it is reasonable to attribute coherent brain changes across participants to fluctuations in cognitive, sensory, or emotional state induced by the stimulus. Such fluctuating but consistent patterns of activity also covary with coherent stimulus-induced intersubject physiological fluctuations (e.g., heart rate-variability; Nguyen, Breakspear, Hu, & Guo, 2016).

The naturalistic stimuli and intersubject correlation paradigm can be extended beyond single brain regions to estimate patterns of interregional FC that show synchronized fluctuations across individuals (intersubject functional connectivity). A growing number of studies have observed synchronized fluctuations in TVFC as participants watch a movie or listen to a story (Betzel, Byrge, Esfahlani, & Kennedy, 2019; Bolton, Jochaut, Giraud, & Van De Ville, 2019; Manning et al., 2018; Simony et al., 2016), and that these fluctuations can be reliably tied to narrative elements of the story (Betzel et al., 2019; Manning et al., 2018; Simony et al., 2016). These data provide further evidence that TVFC methods can reveal subtle fluctuations in cognitive state and suggest that variation in ongoing cognitive processes during task-free conditions can modulate the temporal structure of FC.

##### BOLD TVFC and “spontaneous” cognition during the resting state.

The absence of task instruction or experimentally controlled sensory stimulation does not imply the absence of ongoing cognition. Identifying the physiological and neural markers of ongoing fluctuations in cognitive and emotional states remains one of the core goals of cognitive neuroscience, and the application of BOLD TVFC analyses to these questions has the potential to provide new mechanistic insights.

Though it is well established that cognitive states fluctuate over short timescales, it remains unclear to what extent these fluctuations might be reflected in BOLD TVFC. As highlighted in previous sections, a broad consensus has emerged that there exist robust, reliable differences in BOLD activity and FC between different externally cued cognitive tasks. However, compared with task-based studies, the effect of “spontaneous” fluctuations in mental state on brain activity and FC may be relatively small, and there are concerns about the extent to which such changes can be observed in BOLD TVFC (Kucyi, Tambini, Sadaghiani, Keilholz, & Cohen, 2018; see the section on stability of functional connectivity networks, below, for further discussion of this concern). Attempts to study the content, quality, and dynamics of “spontaneous” cognition—which occur on unpredictable and uncontrolled timescales—also pose a significant experimental challenge. “On-line” measurement methods such as thought probes and experience sampling can provide relatively frequent self-reports of mental state (Christoff, Gordon, Smallwood, Smith, & Schooler, 2009; Kucyi & Davis, 2014; Kucyi et al., 2016) but at the risk of inducing an “observer effect” through demand characteristics that could potentially influence the cognitive processes under study (e.g., by introducing an implicit task demand of metacognitive monitoring). Retrospective reports (e.g., post-scan questionnaires; Delamillieure et al., 2010; Diaz et al., 2013; Gorgolewski et al., 2014) avoid this potential pitfall, but at the cost of significantly reduced temporal resolution.

Despite these challenges, many fundamental questions about the relationship between TVFC and ongoing cognition necessitate the application of these methods, and there exists an emerging literature demonstrating their feasibility and utility (Kucyi, Tambini, et al., 2018). Multiple studies suggest that resting BOLD TVFC is associated with a variety of “spontaneous” cognitive processes, including self-reported stimulus-independent thoughts (i.e., mind wandering; Chou et al., 2017; Kucyi & Davis, 2014; Kucyi et al., 2013; Mittner et al., 2014) and fluctuations in arousal, vigilance, and perceptual performance (Sadaghiani et al., 2015; Shine, Bissett, et al., 2016; G. J. Thompson, Magnuson, et al., 2013; C. Wang et al., 2016). Although these studies did not all include “pure rest” conditions, the types of cognitive processes they investigated are all likely to fluctuate during typical wakeful rest. The extent to which such processes are separable from one another remains an open question, but these methods can be complemented by simultaneous recording of physiological signals (e.g., eye tracking, cardiac and respiratory monitoring) to assess the extent to which observed changes in TVFC are driven mainly by the content/quality of cognition versus concomitant physiological processes.

### Reasons for Skepticism Regarding the Cognitive Relevance of Resting BOLD TVFC

While there is a rapidly growing literature on the cognitive relevance of resting BOLD TVFC, there remain reasons for skepticism. Below, we review three lines of evidence that raise important questions about which cognitive and physiological factors drive observations of resting TVFC, as well as the extent to which functional brain networks reconfigure in response to changes in cognition and behavior.

##### TVFC during anesthesia.

Some of the strongest evidence against the cognitive relevance of BOLD TVFC comes from studies that have shown that TVFC is present during unconsciousness due to general anesthesia (Barttfeld et al., 2015; Hutchison, Womelsdorf, Gati, et al., 2013; Liang, Liu, & Zhang, 2015), when one should expect no changes in cognitive state or level of arousal. Indeed, a number of studies that helped to establish a neural basis for BOLD TVFC made use of simultaneous electrophysiological and fMRI recordings from anesthetized animals (e.g., G. J. Thompson, Merritt, et al., 2013). These results suggest that at least some fraction of BOLD TVFC must be reflective of noncognitive intrinsic or homeostatic processes. That said, studies have also found differences in the characteristics of TVFC observed during wakefulness versus anesthesia. For example, there appear to be a larger repertoire of transient functional connectivity states observed when monkeys are awake than when sedated, including more anticorrelations and FC configurations that deviate from the underlying structural connectivity (Barttfeld et al., 2015). Similarly, brain activity in rats undergoing progressive levels of anesthesia visits fewer distinct states and exhibits fewer transitions as anesthesia deepens (Hudetz, Liu, & Pillay, 2015; Hutchison, Hutchison, Manning, Menon, & Everling, 2014; Y. Ma, Hamilton, & Zhang, 2017). While these studies demonstrate that some aspects of TVFC in the awake condition are distinct from the anesthetized condition, other TVFC properties they measured were remarkably similar between conditions. Sedated animals still displayed state switching patterns, and the duration spent in each state when anesthetized was comparable to the awake condition. A key challenge going forward will be to determine which TVFC properties (if any) are specifically sensitive to fluctuations in cognitive state rather than the kind of intrinsic brain dynamics present during unconsciousness.

##### Confounding by head motion.

Head motion during scanning is likely one of the most significant confounding factors influencing the estimation of BOLD functional connectivity (Power, Barnes, Snyder, Schlaggar, & Petersen, 2012; Van Dijk, Sabuncu, & Buckner, 2012), and numerous papers have demonstrated that even very small amounts of head motion can result in biased estimates of static FC (Power, Schlaggar, & Petersen, 2015; Satterthwaite et al., 2012). Some recent work has suggested that head motion may lead to similarly biased estimates of TVFC, and that current tools to reduce these effects may not be sufficiently effective (Laumann et al., 2017). In contrast, other studies suggest that head motion may have only a small impact on TVFC measures and their reliability (Abrol et al., 2017). There is a pressing need for additional work in this area to help establish a more robust understanding of the extent to which different aspects of BOLD TVFC are influenced by head motion, and to identify effective data-cleaning strategies.

##### Stability of functional connectivity networks.

Much of the interest in rfMRI FC over the past two decades has been due to its high reliability. Resting static FC can provide a robust estimate of an individual’s functional network architecture that is stable across multiple scans and imaging sessions, even months or years apart (Guo et al., 2012; Shehzad et al., 2009; J. H. Wang et al., 2011). Studies have also shown that static FC networks observed during task and rest are highly similar, correlating at up to *r* = 0.9 (Calhoun et al., 2008; Cole et al., 2014; Geerligs et al., 2015; Gratton et al., 2018), and that patterns of static FC are relatively stable across different tasks, with similarity estimates ranging from *r* = 0.5 to *r* = 0.9 (Cole et al., 2014; Finn et al., 2017; Geerligs et al., 2015; Gratton et al., 2018; Krienen, Yeo, & Buckner, 2014). Taken together, these studies suggest that although they are behaviorally significant and reliably observed (Shine & Poldrack, 2018), reconfigurations of static BOLD FC patterns across cognitive states may be relatively subtle, and that small changes to a largely stable underlying functional network architecture may be sufficient to produce a wide variety of cognitive and behavioral states. In light of this evidence that variation in static FC network structure is small even between behaviorally distinct states (e.g., task vs. rest), some researchers have expressed skepticism about whether endogenously driven fluctuations in resting cognition will have any observable influence on BOLD FC. These concerns underscore the importance of developing and utilizing statistically robust TVFC methods and effective data-cleaning strategies. In their absence, researchers seeking to demonstrate the cognitive relevance of resting TVFC will have great difficulty convincing skeptics that observations of resting TVFC are due to ongoing cognitive processes rather than confounds such as head motion or sampling variability.

### Future Directions in Studying the Cognitive Relevance of BOLD TVFC

Given the lack of consensus as to the cognitive relevance of resting BOLD TVFC, it is clear that more work is needed to adjudicate this controversy. While there are many different ways to approach this problem, here we focus on two approaches that we feel are of particular interest and utility.

##### Continuous task paradigms.

Many studies of task-evoked changes in functional connectivity explicitly test for differences in FC between two or more cognitive states that are either imposed by the experimental paradigm or inferred from post hoc analysis of behavioral performance. Often, these states are split across multiple scans. However, a growing number of studies make use of continuous task paradigms in which task demands change over the course of a single scan (Gonzalez-Castillo et al., 2019; Gonzalez-Castillo et al., 2012; Xie et al., 2017). Data from such experiments can provide a powerful opportunity to test the sensitivity of methods for estimating BOLD TVFC. Given an fMRI time series that spans multiple cognitive or behavioral states (as imposed by the experimenter or inferred from analysis of behavior), the validation question becomes whether our statistical methods are able to identify—directly from the fMRI data—fluctuations in BOLD FC that track the experimentally imposed (or behaviorally defined) conditions. Methods that show promise in reliably identifying TVFC fluctuations that align with known shifts in cognition and behavior will be best suited for studying BOLD TVFC at rest, where states and transitions are not known a priori.

##### Causal manipulation of TVFC via brain stimulation.

Brain stimulation technology is evolving rapidly, and recent years have seen the development of methods such as transcranial electrical stimulation—tACS (Ali, Sellers, & Frohlich, 2013; Helfrich et al., 2014) and tDCS (Keeser et al., 2011; Polania, Nitsche, & Paulus, 2011; Polania, Paulus, & Nitsche, 2012; Sehm et al., 2012)—and rhythmic TMS (Hanslmayr, Matuschek, & Fellner, 2014; Romei, Thut, & Silvanto, 2016; Thut, Schyns, & Gross, 2011; Thut, Veniero, et al., 2011) that may allow for the experimental modulation of interregional neural synchrony. While some controversy remains as to the efficacy and reliability of these methods (Lafon et al., 2017; Voroslakos et al., 2018), they potentially provide an exciting opportunity for researchers interested in TVFC. It may soon be possible to dynamically modulate patterns of FC in human subjects while they sit quietly at rest or engage in an experimental task. Changes in cognitive state or behavioral performance that reliably track experimentally modulated FC would provide causal evidence for the cognitive and behavioral relevance of TVFC. Complementary work using simultaneous brain stimulation and fMRI could help to further uncover the precise neural basis of BOLD TVFC, for example by varying stimulation across frequency bands and measuring at which frequencies stimulation has the greatest impact on estimates of BOLD TVFC. In this vein, recent work using direct intracranial brain stimulation in neurosurgical patients (single pulse stimulation to measure cortico-cortical-evoked potentials in target regions) has begun exploring the relationship between the organization of BOLD FC networks and stimulation-evoked responses (Keller et al., 2011; Shine et al., 2017), and we expect that these and similar methods will see increased use in the coming years.

## CLEANING DATA FOR TVFC ANALYSIS

Preprocessing plays a critical role in removing confounds (e.g., head motion, cardiac and respiratory signals) prior to analysis of fMRI data, and this is particularly true when working with data acquired during rest. While most standard rfMRI preprocessing steps (Caballero-Gaudes & Reynolds, 2017) are applicable to TVFC analyses, certain steps require that special care be taken. It has recently been observed that although nuisance correction occurs prior to TVFC estimation, correlations with nuisance regressors can reemerge once TVFC has been estimated (Nalci, Rao, & Liu, 2019). For sliding-window analyses, given a window of length *ω*, it has been recommended to remove frequency components below 1/*ω* (Leonardi & Van De Ville, 2015), although recent work suggests shorter windows may provide improved accuracy when using TVFC estimates to predict phenotypic characteristics (V. M. Vergara et al., 2017; Zalesky & Breakspear, 2015). Finally, there is evidence that time series autocorrelation may influence TVFC estimates, and that removing the effects of autocorrelation through prewhitening of the data may decrease sampling variability (Lehmann et al., 2017). Together, these findings suggest that TVFC-specific nuisance cleaning pipelines may be needed to optimally remove the effect of motion and other signals of noninterest (Lehmann et al., 2017; Lydon-Staley, Ciric, Satterthwaite, & Bassett, 2019; V. M. Vergara et al., 2017), and that the impact of denoising strategy should be considered when interpreting the results of BOLD TVFC analyses and when comparing findings across studies.

In addition to preprocessing, when comparing TVFC measures between groups it is important to undertake control analyses to ensure that observed differences in TVFC are not driven by systematic artifacts (e.g., group differences in head motion). This can be done by directly comparing potential confounding factors between groups, or by testing for a correlation between potential confounds and TVFC. It is also possible to test for residual confounding after preprocessing and compare these residual estimates between groups (Ciric et al., 2017; Parkes, Fulcher, Yucel, & Fornito, 2018).

### Minimizing the Influence of Head Motion

A number of recent studies have attempted to benchmark the effectiveness of different pipelines for mitigating the effects of head motion on estimates of BOLD FC (Ciric et al., 2017; Parkes et al., 2018). Consistent results suggest that pipelines that include independent component analysis denoising and global signal regression (GSR) may be most effective. However, to our knowledge, only one study has so far attempted a systematic comparison of motion denoising pipelines specifically for TVFC analyses (Lydon-Staley et al., 2019). As is the case for static FC, motion contamination of TVFC estimates was minimized most effectively by pipelines that included GSR, though it should be noted that in general the use of GSR is somewhat controversial (Murphy & Fox, 2017). It is important that this new work be replicated and expanded upon to begin building a consensus on optimal data-cleaning strategies for TVFC analyses.

Volume censoring (“scrubbing”; Power et al., 2012) is another commonly used method to minimize the effects of head motion on FC estimates. By removing volumes with high levels of apparent motion, the issue of motion contamination is essentially sidestepped. Censoring can be very effective in studies of static FC (e.g., Power et al., 2014), and recent work suggests that it may be similarly powerful in the context of TVFC (Laumann et al., 2017). However, because censoring disrupts the temporal relationship between time points, it can interact in undesirable ways with subsequent TVFC analyses that implicitly or explicitly consider these factors. For example, censoring prior to sliding-window analysis can result in windows that contain unequal numbers of time points (Hutchison, Womelsdorf, Allen, et al., 2013; Zalesky et al., 2014), and time series with uneven spacing between time points due to censoring are incompatible with most time-frequency-based approaches.

### Accounting for Arousal-Related Effects

As discussed in depth above, several studies have shown that changes in vigilance and arousal can impact measures of brain function. While the ability to detect changes in arousal can be considered a strength of TVFC, it can also be considered a potential confound. Studies that wish to make inferences about the cognitive relevance of TVFC (or compare TVFC between groups) independent of arousal must therefore measure and account for the presence and influence of these fluctuations. Although it can be difficult to track subtle fluctuations in arousal, measurements of pupil diameter (Schneider et al., 2016; Shine, Bissett, et al., 2016) or eyelid closure (Allen et al., 2018; Chang et al., 2016; C. Wang et al., 2016) can provide an independent physiological index of these fluctuations. Methods have also been proposed that estimate levels of arousal directly from fMRI data (Haimovici et al., 2017; Tagliazucchi & Laufs, 2014). Whether fluctuations in arousal are an interesting source of neural variation ultimately depends on the specific research question.

There is currently no consensus on the optimal way to account for the effects of fluctuations in arousal in the context of TVFC analyses. One relatively simple approach that has been used with success in studies of static FC is to discard time points during which participants are asleep or have very low levels of arousal (J. Wang, Han, Nguyen, Guo, & Guo, 2017). However, as mentioned above, censoring strategies are often undesirable for studies of TVFC, as they disrupt the spacing and ordering of volumes and preclude certain types of TVFC analyses. Instead, the influence of fluctuating arousal can be dealt with statistically by regressing out continuous measures of arousal (such as those mentioned in the previous paragraph). While careful experimental design (e.g., real-time vigilance monitoring) and the use of statistical models can be used to try to minimize the effects of sleep and arousal, doing so is often nontrivial as one needs a very well-articulated model of the contribution of the confounding sources in order to account for them. More work is needed to further characterize the impact of arousal on BOLD TVFC and to identify effective strategies to account for these effects.

Finally, we emphasize that there is an inherent trade-off when attempting to remove the influence of arousal on BOLD TVFC: These processing steps may very likely remove interesting signal along with putative sources of noise, as cognitive processes are often intricately linked with arousal and other physiological states. It will be important for future work in the field to appropriately disambiguate the arousal-related signatures that are either detrimental to or facilitative of cognitive performance, thus refining our understanding of the building blocks of the brain’s cognitive architecture.

## WHAT CAN WE CONCLUDE, AND HOW SHOULD WE THINK ABOUT RESTING BOLD TVFC?

Given the evidence we have reviewed, what conclusions can we draw regarding the three questions posed at the beginning of this article? First, we believe that when applied to properly cleaned data, a diverse landscape of analytic and modeling approaches are capable of reliably estimating TVFC from BOLD rfMRI time series. As to whether estimates of time-resolved functional connectivity observed during rest truly vary in time, we emphasize that the space of “dynamic” phenomena is large, and that the answer to this question depends critically on selecting appropriate time series features and null models for the specific hypothesis being tested. Second, a robust literature on the physiological basis of static BOLD FC and TVFC suggests that these phenomena result in large part from interregional synchronization of neural activity, and that patterns of synchronization can be modulated by level of arousal. Third, there is a broad consensus that external task demands can modulate patterns of BOLD FC, and growing evidence that TVFC fluctuations during rest are not only reliable within and across individuals, but relevant to ongoing cognition and behavior.

Beyond the questions that inspired this review, it is worth taking a step back and considering how TVFC phenomena relate to traditional static FC. How should we think about the transient patterns of FC identified by TVFC methods? It seems likely that we should expect to see both similarities and differences in TVFC results relative to patterns of static FC. We might expect similarities based on evidence that static FC states often represent behaviorally relevant functional network configurations, and it is reasonable to expect that these same configurations may be briefly recapitulated at faster timescales. We may expect to see differences because given FC that varies in time, patterns of static FC will essentially capture some aggregate measure of the strength of a functional connection over the window in which static FC is calculated. Like any summary measure, these aggregate static FC estimates will likely fail to capture some aspects of the underlying TVFC.

This is an important point, because it can potentially help address the paradox of FC stability (discussed above): Given that patterns of static FC are so similar across a wide range of behavioral states, why should we expect to see fluctuations in FC during periods of rest, when overt behavior remains unchanged? The key to resolving this paradox may involve recognizing that a given value of a summary measure can be realized by multiple different arrangements of the underlying data. In the context of FC, this means that the same pattern of static FC may result from different spatiotemporal patterns of underlying TVFC. Further, because TVFC fluctuations unfold over time in a particular order, the same distribution of TVFC patterns (e.g., 4 TRs in pattern A, 4 TRs in pattern B) can have very different temporal profiles (ABABABAB, AAAABBBB, AABBAABB, etc.). Brain dynamics unfold over time in a particular sequence, and it is therefore important to go beyond simply identifying FC patterns at high temporal resolution: To further our understanding of brain dynamics, cognition, and behavior, we must also consider the temporal aspects of TVFC fluctuations (e.g., transition probabilities, dwell times, switching rates). It is not enough to know *what* FC patterns occur, but also *when*. This idea features prominently in recent work on the temporal profiles (metastates) of TVFC during rest (Vidaurre et al., 2017), and how these profiles differ between wake and sleep (Damaraju et al., 2018; Stevner et al., 2019). From this perspective, there may be no paradox at all: Fluctuations in resting TVFC are not necessarily inconsistent with stable, static FC.

## ADVANCING THE FIELD: RECOMMENDATIONS FOR MOVING FORWARD

It is our hope that this paper can serve as not only a review of the current state of the field, but also a blueprint for future work. TVFC analyses of BOLD fMRI and other types of neuroimaging data have the potential to help answer some of the most compelling open questions in cognitive and systems neuroscience. TVFC analyses of intrinsic brain activity recorded at rest are fast becoming a key tool for researchers seeking to identify fundamental principles of macroscale brain dynamics, their spatial and temporal organization, and their relationship to underlying anatomy. Studies of resting TVFC have also begun to shed light on disordered intrinsic brain dynamics in individuals with psychiatric and neurological illness, and careful experiments using online measures and naturalistic paradigms promise to reveal fine-grained relationships between patterns of functional connectivity and cognitive, behavioral, and physiological states. At the same time, important questions remain unresolved. How much variance in resting TVFC is explainable by various contributing factors (e.g., neural signaling, bodily physiology, cognitive state, apparent head motion)? Precisely how sensitive is BOLD TVFC to shifts in cognition? Can we resolve “spontaneous” changes in mental content (e.g., visualizing a place vs. a face), or are we limited to studying more general changes in cognitive state (e.g., goal-directed future planning vs. undirected mind wandering)? Success in answering these questions will require contributions from and collaboration between researchers with a wide range of backgrounds and perspectives. With this in mind, we offer the following concrete recommendations aimed at facilitating a consensus approach for research into time-varying functional connectivity.

First, we urge researchers undertaking TVFC analyses to carefully consider their choice of terminology when describing their methods and framing their results. Inconsistencies in definitions between researchers have the potential to needlessly muddy an already complicated scientific landscape. While we have proposed the term “time-varying functional connectivity” as an appropriately broad label, we recognize that debates about the application of this and other terms are likely to continue. Beyond the specific case of TVFC, there is also ongoing debate about the use of “functional connectivity” to refer to methods that attempt to infer neural interactions from time series data (Mehler & Kording, 2018; Reid et al., 2019). As the field evolves, the terminology will undoubtedly continue to expand along with it. This underscores the importance of ensuring we are clear about our terms and definitions, and that we consider their use in the context of existing terminology.

Second, while TVFC methods provide an opportunity to address many interesting questions, they must be carefully applied. Care must be taken in preprocessing and the selection of the appropriate analytic approaches and null models. Findings should be appropriately contextualized against the backdrop of potentially conflicting evidence regarding the validity and reliability of BOLD TVFC methods. Experimentalists, theorists, and quantitative methodologists must continue to work together to identify and communicate best practices to help ensure a reliable and useful literature on BOLD TVFC. We encourage those new to TVFC analyses to take the time to learn and understand the peculiarities and pitfalls of these methods, and to engage in discussions with domain experts to ensure that their findings are robust.

Third, we propose that future work on BOLD TVFC be considered from the perspective of the three key questions we outline at the beginning of this article. While the questions we raise are essentially sequential (e.g., there’s little value in considering the biological basis of TVFC if we conclude that resting BOLD data do not contain TVFC in the first place), we recognize that science rarely proceeds in an orderly fashion. As such, it is critical that studies exploring the “latter” questions make clear on which untested or controversial assumptions they rest (e.g., that resting BOLD data exhibit TVFC, are the result of underlying neural dynamics).

As the field continues to move forward, the study of resting brain dynamics will benefit from both the refinement of existing TVFC methodologies as well as the use and development of complementary techniques. For example, methods capable of recovering the hemodynamic response function from rfMRI data promise to further elucidate the relationship between neural activity at rest and the observed BOLD signal. This knowledge can in turn help inform the development of models that facilitate the estimation of time-varying directed or “effective” functional connectivity. As our tools and analyses continue to develop, it will also be critical to assess the impact of data quality and quantity (e.g., MRI sequence parameters, scan duration, number of participants) on individual and group-average TVFC estimates.

Overall, we believe that statistically rigorous, well-validated studies of resting BOLD TVFC have the potential to greatly expand our understanding of brain dynamics and their relationship to cognition in health and disease, and that collaborative, open work towards resolving outstanding controversies is the most effective and productive path forward for our field.

## ACKNOWLEDGMENTS

We would like to thank Cesar Caballero-Gaudes, Peter Fransson, Lucina Uddin, Sepideh Sadaghiani, Daniele Marinazzo, and Ioannis Pappas for their comments and suggestions on earlier versions of this manuscript. We also thank Abraham Snyder, Jean-Baptiste Poline, Ted Satterthwaite, Caterina Gratton, and Nico Dosenbach for their participation in the discussion that inspired us to write this review.

## SUPPORTING INFORMATION

Supporting information for this article is available at https://doi.org/10.1162/netn_a_00116. Python code for creating Figure 1 and components of Figure 2 can be found at Lurie (2019).

## AUTHOR CONTRIBUTIONS

Daniel J. Lurie: Conceptualization; Project administration; Supervision; Visualization; Writing - Original Draft; Writing - Review & Editing. Daniel Kessler: Conceptualization; Project administration; Supervision; Writing - Original Draft; Writing - Review & Editing. Danielle S. Bassett: Writing - Original Draft; Writing - Review & Editing. Richard F. Betzel: Writing - Original Draft; Writing - Review & Editing. Michael Breakspear: Writing - Original Draft; Writing - Review & Editing. Shella Kielholz: Writing - Original Draft; Writing - Review & Editing. Aaron Kucyi: Writing - Original Draft; Writing - Review & Editing. Raphaël Liégeois: Writing - Original Draft; Writing - Review & Editing. Martin A. Lindquist: Writing - Original Draft; Writing - Review & Editing. Anthony Randal McIntosh: Writing - Original Draft; Writing - Review & Editing. Russel A. Poldrack: Writing - Original Draft; Writing - Review & Editing. James M. Shine: Writing - Original Draft; Writing - Review & Editing. William Hedley Thompson: Writing - Original Draft; Writing - Review & Editing. Natalia Z. Bielczyk: Visualization; Writing - Review & Editing. Linda Douw: Writing - Review & Editing. Dominik Kraft: Writing - Review & Editing. Robyn L. Miller: Writing - Review & Editing. Muthuraman Muthuraman: Writing - Review & Editing. Lorenzo Pasquini: Writing - Review & Editing. Adeel Razi: Writing - Review & Editing. Diego Vidaurre: Writing - Review & Editing. Hua Xie: Writing - Review & Editing. Vince D. Calhoun: Conceptualization; Project administration; Supervision; Writing - Original Draft; Writing - Review & Editing.

## FUNDING INFORMATION

Daniel J. Lurie, National Science Foundation, Award ID: DGE 1106400. Daniel J. Lurie, National Institute of Neurological Disorders and Stroke, Award ID: 1F31NS108665-01A1. Daniel Kessler, National Science Foundation, Award ID: DMS-1646108. Danielle S. Bassett, John D. and Catherine T. MacArthur Foundation. Danielle S. Bassett, Alfred P. Sloan Foundation. Danielle S. Bassett, ISI Foundation. Danielle S. Bassett, Paul Allen Foundation. Danielle S. Bassett, Army Research Laboratory, Award ID: W911NF-10-2-0022. Danielle S. Bassett, Army Research Office, Award ID: Bassett-W911NF-14-1-0679. Danielle S. Bassett, Army Research Office, Award ID: Grafton-W911NF-16-1-0474. Danielle S. Bassett, Army Research Office, Award ID: DCIST-W911NF-17-2-0181. Danielle S. Bassett, Office of Naval Research. Danielle S. Bassett, National Institute of Mental Health, Award ID: 2-R01-DC-009209-11. Danielle S. Bassett, National Institute of Mental Health, Award ID: R01-MH112847. Danielle S. Bassett, National Institute of Mental Health, Award ID: R01-MH107235. Danielle S. Bassett, National Institute of Mental Health, Award ID: R21-M MH-106799. Danielle S. Bassett, National Institute of Child Health and Human Development, Award ID: 1R01HD086888-01. Danielle S. Bassett, National Institute of Neurological Disorders and Stroke, Award ID: R01 NS099348. Danielle S. Bassett, National Science Foundation, Award ID: BCS-1441502. Danielle S. Bassett, National Science Foundation, Award ID: BCS-1430087. Danielle S. Bassett, National Science Foundation, Award ID: NSF PHY-1554488. Danielle S. Bassett, National Science Foundation, Award ID: BCS-1631550. Richard F. Betzel, Indiana University Office of the Vice President for Research, Award ID: Emerging Area of Research Initiative, Learning: Brains, Machines, and Children. Michael Breakspear, National Health and Medical Research Council, Award ID: 118153. Michael Breakspear, National Health and Medical Research Council, Award ID: 10371296. Michael Breakspear, National Health and Medical Research Council, Award ID: 1095227. Michael Breakspear, Australian Research Council, Award ID: CE140100007. Shell Kielholz, National Institutes of Health, Award ID: R01MH111416. Shell Kielholz, National Institutes of Health, Award ID: R01NS078095. Shell Kielholz, National Science Foundation, Award ID: BCS INSPIRE 1533260. Aaron Kucyi, Canadian Institutes of Health Research, Award ID: Banting Fellowship. Raphaël Liégeois, CHIST-ERA (http://dx.doi.org/10.13039/501100001942), Award ID: IVAN 20CH21 174081. Martin A. Lindquist, National Institute of Biomedical Imaging and Bioengineering, Award ID: R01 EB016061. Martin A. Lindquist, National Institute of Biomedical Imaging and Bioengineering, Award ID: R01 EB026549. James M. Shine, National Health and Medical Research Council, Award ID: GNT1072403. James M. Shine, National Health and Medical Research Council, Award ID: GNT1156536. William Hedley Thompson, Knut and Alice Wallenberg Foundation, Award ID: 2016.0473. Linda Douw, Society in Science, Award ID: Branco Weiss Fellowship. Linda Douw, Dutch Organization for Scientific Research, Award ID: 016.146.086. Adeel Razi, Australian Research Council, Award ID: DECRA, DE170100128. Dominik Kraft, German Research Foundation, Award ID: CRC 1193. Dominik Kraft, German Research Foundation, Award ID: INST 247/859-1. Muthuraman Muthuraman, German Research Foundation, Award ID: SFB CRC-1193. Muthuraman Muthuraman, German Research Foundation, Award ID: SFB CRC-TR-128. Vince D. Calhoun, National Institutes of Health, Award ID: R01EB020407. Vince D. Calhoun, National Institutes of Health, Award ID: P20GM103472. Vince D. Calhoun, National Institutes of Health, Award ID: P30GM122734. Vince D. Calhoun, National Science Foundation, Award ID: 1539067.

## TECHNICAL TERMS

Arousal: A continuous property of brain and bodily states that influences information processing and behavioral responsivity though effects on neural signaling.
“Resting” state: Behavioral state with minimal (or no) explicit task demands. Participants may engage in a wide range of self-directed cognitive processes.
Sampling variability: For a given distribution, how much a computed statistic varies across samples (e.g., time points, scans, or participants).
Neuromodulator: A chemical thatacts to modulate the function of a neuron (e.g., by increasing or decreasing input gain).
Naturalistic stimuli: Complex stimuli (e.g., movies, podcasts, video games) that better approximate everyday cognitive and perceptual experience than simplified experimental paradigms.
