# Supplementary material for: Questions and controversies in the study of time-varying functional connectivity in resting fMRI
Source: Netw Neurosci. 2020 Feb 1;4(1):30–69. doi: 10.1162/netn_a_00116 (PMC7006871; doi:10.1162/netn_a_00116)
Supplement: Supplementary file 1 [file netn-04-30-s001.pdf]

Lurie, D. J., Kessler, D., Bassett, D. S., Betzel, R. F., Breakspear, M., Keilholz, S., Kucyi, A…Calhoun, V. D., (2020). Supporting information for "Questions and controversies in the study of time-varying functional connectivity in resting fMRI." *Network Neuroscience*, *4*(1), 30-69. https://doi.org/10.1162/netn_a_00116

Supplementary Information:

Publication counts for Figure 1 were determined by searching the NIH PubMed database.

The search term for all TVC papers was:

```
("dynamic connectivity"[TIAB] OR "dynamic functional connectivity"[TIAB] OR "dynamic FC"[TIAB] OR "connectivity dynamics"[TIAB] OR "FC dynamics"[TIAB] OR "functional network dynamics"[TIAB] OR "FC network dynamics"[TIAB] OR "functional brain network dynamics"[TIAB] OR "time-varying connectivity"[TIAB] OR "time-varying functional connectivity"[TIAB] OR "time-varying FC"[TIAB] OR "time varying connectivity"[TIAB] OR "time varying functional connectivity"[TIAB] OR "time varying FC"[TIAB] OR "time resolved connectivity"[TIAB] OR "time resolved functional connectivity"[TIAB] OR "time resolved FC"[TIAB] OR "time-resolved connectivity"[TIAB] OR "time-resolved functional connectivity"[TIAB] OR "time-resolved FC"[TIAB] OR "dynamic functional network connectivity"[TIAB]) AND ("functional magnetic resonance imaging"[TIAB] OR "functional MRI"[TIAB] OR "fmri"[TIAB])
```

The search term for resting TVC papers was:

```
("dynamic connectivity"[TIAB] OR "dynamic functional connectivity"[TIAB] OR "dynamic FC"[TIAB] OR "connectivity dynamics"[TIAB] OR "FC dynamics"[TIAB] OR "functional network dynamics"[TIAB] OR "FC network dynamics"[TIAB] OR "functional brain network dynamics"[TIAB] OR "time-varying connectivity"[TIAB] OR "time-varying functional connectivity"[TIAB] OR "time-varying FC"[TIAB] OR "time varying connectivity"[TIAB] OR "time varying functional connectivity"[TIAB] OR "time varying FC"[TIAB] OR "time resolved connectivity"[TIAB] OR "time resolved functional connectivity"[TIAB] OR "time resolved FC"[TIAB] OR "time-resolved connectivity"[TIAB] OR "time-resolved functional connectivity"[TIAB] OR "time-resolved FC"[TIAB] OR "dynamic functional network connectivity"[TIAB]) AND ("functional magnetic resonance imaging"[TIAB] OR "functional MRI"[TIAB] OR "fmri"[TIAB]) AND ("rest"[TIAB] OR "resting"[TIAB] OR "resting-state"[TIAB] OR "intrinsic"[TIAB])
```

Python code for creating Figure 1 and components of Figure 2 can be found at:

https://github.com/danlurie/lurie_kessler_2019

Embracing new forums for scientific discourse

Although it is natural to expect that different scientists may have different interpretations of specific empirical findings and the published literature as a whole, efforts to resolve scientific controversies are ultimately a fool's errand if there can be no agreement (or no clarity) on at least the key terms, open questions, and possible adjudicating experiments. While the scholarly literature on TVFC grows richer by the day—and there is no replacement for high quality research outputs—we have found great utility in venues for collaboration and discussion that are more fluid, interactive, and fast-moving than the conventional scientific publication pipeline. Each of these forums have their own unique strengths and weaknesses, but in combination provide an exciting opportunity to facilitate progress on difficult questions in our field and beyond.

Twitter posts and subsequent discussion threads are fantastic for stoking spontaneous, accessible debate among widely distributed researchers. At the same time, these conversations can be challenging to parse after the fact, are difficult to archive, and the arbitrary character constraints often require simplifications that are obfuscating rather than clarifying. Mailing lists trade the spontaneity and real-time interactions of social media for an opportunity to hold conversations in a format that is unconstrained by character limits or the stylistic conventions of journal articles. Their "back-and-forth" structure permits easy archiving and deep, considered dialogue. Offline, conferences offer a unique opportunity for especially meaningful interactions: presentations and the discussions that follow them (including online) afford an opportunity to communicate high-level arguments to a wide audience. We applaud the introduction of panel-style discussions such as those held recently at the 2017 and 2018 Organization for Human Brain Mapping annual meetings. In addition to formal conference sessions, we have seen first-hand the power of ad-hoc in-person discussions to rapidly foster mutual understanding and collaboration on an impressive scale—indeed, this paper is an outgrowth of an open-invitation round-table session organized via Twitter at OHBM 2017[1]. We invite other researchers interested in continuing to advance the discussion around TVFC to join our online discussion group. The "Time-Varying Working Group"[2] offers a forum for continued refinement and updating of key theoretical questions along with discussion of the latest empirical findings.

---

[1] https://twitter.com/danjlurie/status/880201676258332678
[2] https://groups.google.com/forum/#!forum/time-varying-working-group
